# Synthesis, Molecular Docking, Dynamics, Quantum-Chemical Computation, and Antimicrobial Activity Studies of Some New Benzimidazole–Thiadiazole Hybrids

**DOI:** 10.1021/acsomega.2c06142

**Published:** 2022-12-09

**Authors:** Ismail Celik, Ulviye Acar Çevik, Arzu Karayel, Ayşen Işık, Uğur Kayış, Ülküye Dudu Gül, Hayrani Eren Bostancı, Süheyl Furkan Konca, Yusuf Özkay, Zafer Asım Kaplancıklı

**Affiliations:** † Department of Pharmaceutical Chemistry, Faculty of Pharmacy, Erciyes University, 38039 Kayseri, Turkey; ‡ Department of Pharmaceutical Chemistry, Faculty of Pharmacy, Anadolu University, 26470 Eskişehir, Turkey; § Department of Physics, Faculty of Arts and Science, Hitit University, 19030 Çorum, Turkey; ∥ Department of Biochemistry, Faculty of Science, Selçuk University, 42250 Konya, Turkey; ⊥ Pazaryeri Vocational School, Program of Pharmacy Services, Bilecik Şeyh Edebali University, 11230 Bilecik, Turkey; # Department of Bioengineering, Faculty of Engineering, Bilecik Şeyh Edebali University, 11230 Bilecik, Turkey; ∇ Department of Pharmaceutical Basic Sciences, Faculty of Pharmacy, Cumhuriyet University, 58140 Sivas, Turkey; ○ Department of Pharmaceutical Biotechnology, Faculty of Pharmacy, Erciyes University, 38039 Kayseri, Turkey

## Abstract

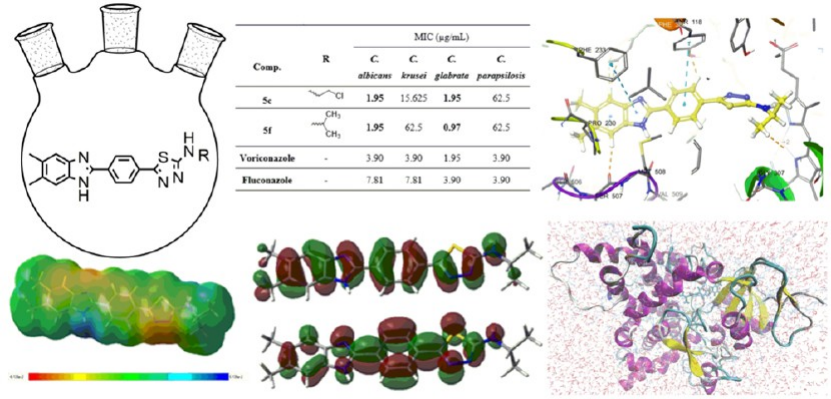

In this study, some new compounds, which are 2-aminothiadiazole derivatives linked by a phenyl bridge to the 2-position of the benzimidazole ring, were designed and synthesized as antimicrobial agents. The structures of the compounds were elucidated by ^1^H and ^13^C NMR spectroscopy, high-resolution mass spectrometry, and elemental analysis. The antifungal activities of the synthesized compounds were tested on *Candida albicans*, *Candida krusei*, *Candida glabrata*, and *Candida parapsilosis*. Compound **5f** is more active against *C. albicans* and *C. glabrata* than standard fluconazole and varicanazole. Compounds were also evaluated for their counteracting activity against Gram-positive *Escherichia coli*, *Serratia marcescens*, *Klebsiella pneumoniae*, and *Pseudomonas aeruginosa* and Gram-negative *Enterococcus faecalis*, *Bacillus subtilis*, and *Staphylococcus aureus*. Compounds **5c** and **5h** had minimum inhibitory concentrations against *E. faecalis* close to that of the standard azithromycin. Molecular docking studies were performed against *Candida* species’ 14-α demethylase enzyme. **5f** was the most active compound against *Candida* species, which gave the highest docking interaction energy. The stabilities of compounds **5c** and **5f** with CYP51 were tested using 100 ns molecular dynamics simulations. According to the theoretical ADME calculations, the profiles of the compounds are suitable in terms of limiting rules. HOMO–LUMO analysis showed that **5h** is chemically more reactive (represented with the lower Δ*E* = 3.432 eV) than the other molecules, which is compatible with the highest antibacterial activity result.

## 1 Introduction

The advancements of modern medicine are being undermined by antibiotic resistance as infectious illnesses become once more serious concern as a result of the global spread of antibiotic-resistant pathogens.^1^ Antimicrobial agents come in a variety of forms, including antibiotics, and can be used to treat microorganisms to decrease their ability to grow, prevent their reproduction, or even kill them. Some substances can significantly change physiological and metabolic processes.^2^ The ability of microorganisms to remain and be alive in the presence of antimicrobial drugs is known as antimicrobial resistance (AMR). Prolonged and extensive use of antibiotics over time has resulted in the development of AMR within microorganisms.^3^ Antimicrobial medicines are used to treat microbial infections, although this type of infection therapy is severely hampered by the development of antibiotic resistance. Therefore, the discovery and development of new antimicrobial drugs is crucial to effectively treat microbial illnesses.^4^ To fight antimicrobial-resistant microorganisms, new therapeutic approaches are considered necessary.

Fungal infection is on the rise around the world, posing a danger to human health and life. Invasive candidiasis, in particular, has risen to become the fourth main reason for bloodstream infection,^5^ causing up to 40% mortality in patients.^6^

*Candida* species are among the most prevalent causes of invasive infections and have distinct properties regarding virulence, antifungal susceptibility, and capability for invasion. Many *Candida* spp. can cause infectious diseases, but the majority are caused by *Candida albicans* and non-*albicansCandida* species like *Candida glabrata*, *Candida krusei*, *Candida parapsilosis*, and *Candida tropicalis*.^7^

Azoles are a significant class of compounds used in the treatment of human, animal, and plant fungus illnesses as well as in the preservation of material. Howver, the widespread application of the same antifungal class in many biotopes does carry a danger since the development of antifungal resistance through use in one field of application may impair the effectiveness of related compounds in other fields of application.^8^ Multiple azole resistance mechanisms in *Candida* species have been identified.^9^ Antifungal medication resistance is characterized as a heritable alteration in the fungus’ sensitivity to medicine or fungicide that decreases the drug’s effectiveness in treating the fungus. During long-term azole therapy, resistance to medical azoles with action against antifungal drugs may develop.^10^ These problems must be overcome for an effective treatment to be carried out.

Thiadiazoles are azole derivatives that are used as structural components of physiologically active compounds and as practical intermediates. Thiadiazole is a heterocyclic nucleus containing sulfur and nitrogen that belongs to a large class of heterocyclic compounds with many isomers that are important in medicine.^11^ The biological activities of molecules containing triazole or thiadiazole are very diverse. Because of the variety of biological activities, 1,3,4 thiadiazole is one of the most beneficial isomeric forms.^12−15^ The 1,3,4-thiadiazole structure is one of the most significant and well-known heterocycles, which is a common and complementary advantage of a range of pharmaceutical and natural substances. The thiadiazole ring is present as a key component in an assemblage of pharmacological categories because 1,3,4-thiadiazole derivatives have a wide range of biological activities, including antimicrobial,^16^ antiviral,^17^ anti-inflammatory, and analgesic,^18^ and anticancer activity.^19^

**Figure 1:**
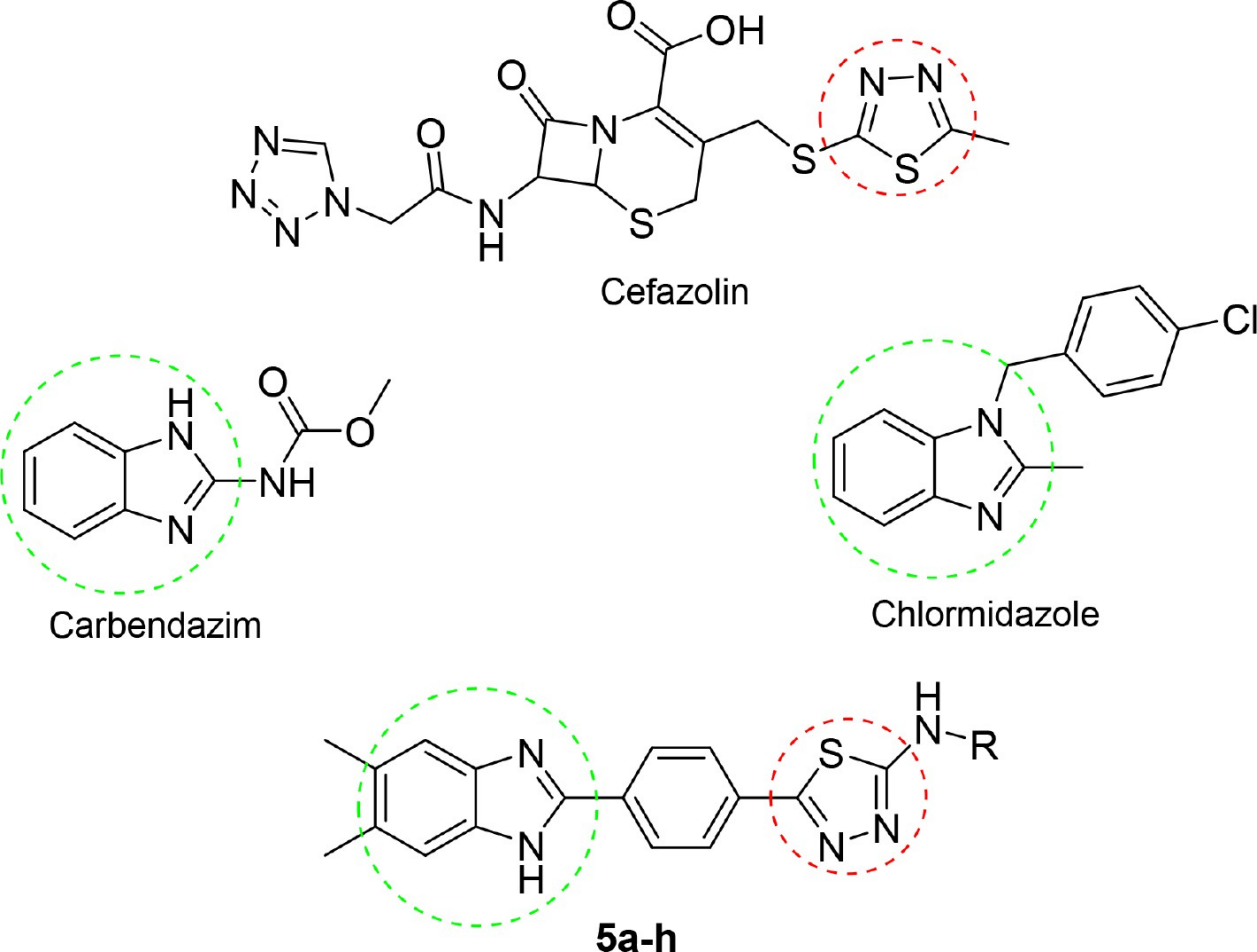
Structures of the designed and synthesized N-substituted 5-(4-(5,6-dimethyl-1*H*-benzimidazol-2-yl)phenyl)-1,3,4-thiadiazol-2-amine derivatives **5a**–**h** as antimicrobial agents, the antibacterial cefazolin, and the antifungals chlormidazole and carbendazim.

In this study, new benzimidazole–thiadiazole hybrids **5a**–**h** were designed by combining the thiadiazole core of the antibacterial cefazolin and the benzimidazole core of the antifungals chlormidazole and carbendazim (the active metabolite of benomyl) (Figure 1). A common method for finding structurally new, powerful molecules with enhanced characteristics is scaffold hopping.^20,21^ In the present study, we adopted the scaffold-hopping strategy^22−24^ and incorporated the benzimidazole group in the thiadiazole moiety to design new antimicrobial scaffold.

Benzimidazole–thiadiazole derivatives were prepared in five steps. The synthesized thiadiazoles were characterized by ^1^H and ^13^C NMR spectroscopy, high-resolution mass spectrometry (HRMS), and elemental analysis and studied as antimicrobial agents against bacterial and fungal species. In addition, molecular docking studies were performed with 14-α demethylase (CYP51) of *Candida*. To examine the stability of compounds with CYP51, 100 ns molecular dynamics (MD) simulations were performed. In the molecular docking study, it is essential to determine the accurate structure of the ligand possessing the lowest minimum energy. For this purpose, the eight newly synthesized compounds were modeled with density functional theory (DFT). Moreover, to understand how electrostatic interactions affect the binding of CYP51 of *Candida* with the newly synthesized benzimidazole–thiadiazole hybrids, molecular electrostatic potential (MEP) and HOMO–LUMO analyses were performed at the B3LYP/6-311G(d,p) level.

## 2 Results and Discussion

### 2.1 Chemistry

**Figure 2:**
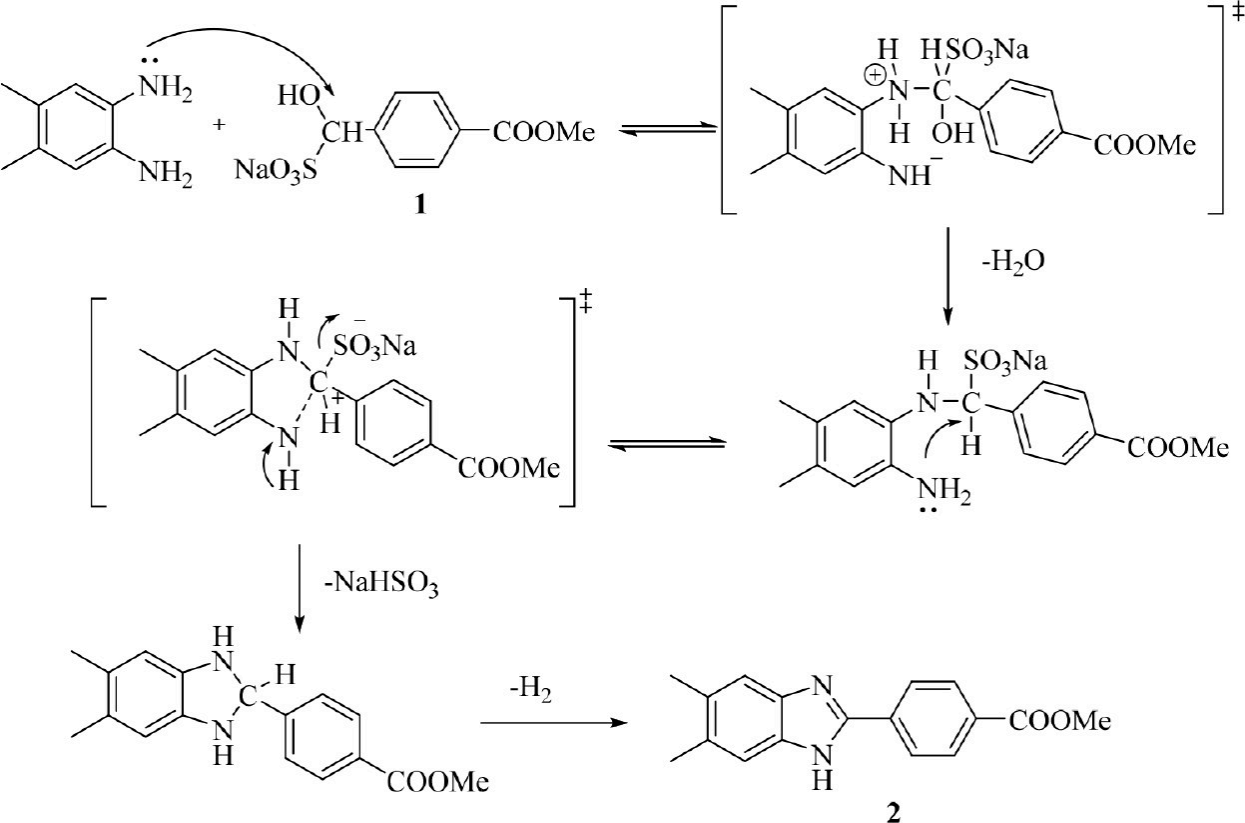
Synthesis pathway for the target 1,3,4-thiazole–benzimidazole hybrid compounds **5a**–**h.** Reagents and conditions: (a) Na_2_S_2_O_5_, EtOH, rt, 1 h; (b) DMF, 12, 5 h; (c) NH_2_NH_2_, EtOH, reflux, 12 h; (d) RNCS, EtOH, reflux, 3 h; (e) H_2_SO_4_, 0 °C, 10 min, then 25 °C, 10 min.

As shown in Figure 2, the target molecules were synthesized in five steps. First of all, the aldehyde part of methyl 4-formylbenzoate was treated with sodium metabisulfite in ethanol to obtain compound **1**, the sodium metabisulfite addition product of the aldehyde. In the second step, as a result of the condensation reaction of **1** and 4,5-dimethyl-1,2-phenylenediamine under reflux, methyl 4-(5,6-dimethyl-1*H*-benzimidazol-2-yl)benzoate (**2**) was obtained. A significant conceptual innovation in organic synthesis is selectivity control, which frequently becomes the bottleneck in the production process. Due to the competitive synthesis of 1,2-disubstituted and 2-substituted benzimidazoles, such a situation has a significant potential to arise during the direct cyclocondensation of *o*-phenylenediamines with aldehydes.^25,26^ With this method, the 2-substituted benzimidazole derivative was obtained selectively.

**Figure 3:**
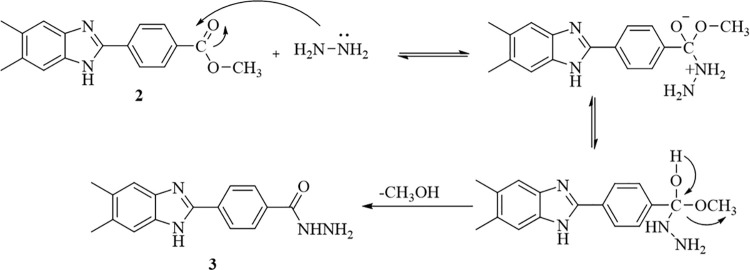
Reaction mechanism for compound **2**.

Based on certain information from the literature, a mechanism has been proposed for the production of benzimidazole **2** (Figure 3). The amine group on the *o*-phenylenediamine makes a nucleophilic assault on the carbon atom of the aldehyde bisulfite compound to start the reaction. There is a loss of one mole of water. The resultant alkylsulfonate then interacts with the other amine group of the *o*-phenylenediamine to produce the intermediate dihydroimidazole. Finally, the benzimidazole ring is obtained by aromatization.^27,28^

**Figure 4:**
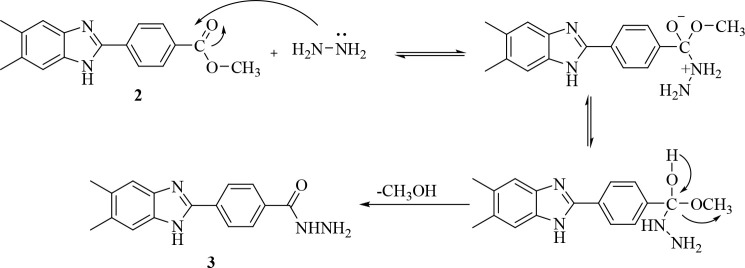
Reaction mechanism for compound **3**.

In the next step, compound **2** was treated with hydrazine hydrate in ethanol to obtain 4-(5,6-dimethyl-1*H*-benzimidazol-2-yl)benzene-1-carbohydrazide (**3**). The proposed mechanism for the reaction of ester derivative compound **2** with hydrazine hydrate to give **3** is given in Figure 4.^29^

**Figure 5:**
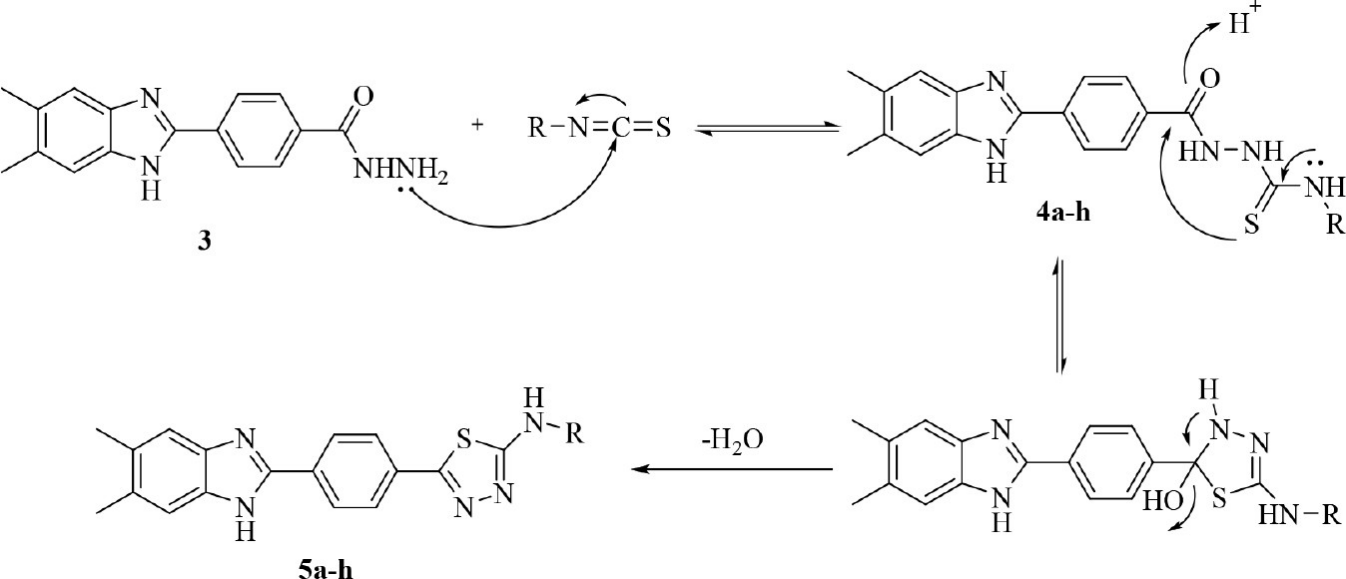
Reaction mechanism for compounds **4a**–**h** and **5a**–**h**.

The hydrazide derivative compound and the appropriate isothiocyanate derivatives in ethanol were refluxed, and the precipitated products were filtered off. In the last step, the thiadiazole derivatives **5a**–**h** were obtained by cyclization of the thiosemicarbazide compound in the presence of concentrated sulfuric acid.^30,31^ The tendency for the 1,3,4-thiadiazole ring to form under acidic conditions may stem from loss of nucleophilicity of N4 as a result of protonation, which then causes a corresponding increase in the sulfur atom’s nucleophilicity toward the attack of the carbonyl carbon. The reaction mechanism for 1,3,4-thiadiazole derivatives **5a**–**h** is shown in Figure 5.^32−34^

### 2.2 Antimicrobial Activity

#### 2.2.1 Antifungal Activity

In vitro antifungal screening of the synthesized compounds was assessed against *C. albicans* ATCC 24433, *C. glabrata* (ATCC 90030), *C. krusei* ATCC 6258, *C. parapsilosis* (ATCC 22019). The minimum inhibitory concentration (MIC) values of the synthesized compounds and the control drugs against fungus strains are summarized in Table 1. Voriconazole and fluconazole were used as reference drugs for antifungal activity.

**Table 1:**
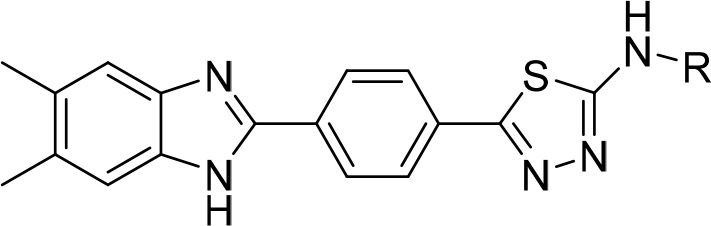

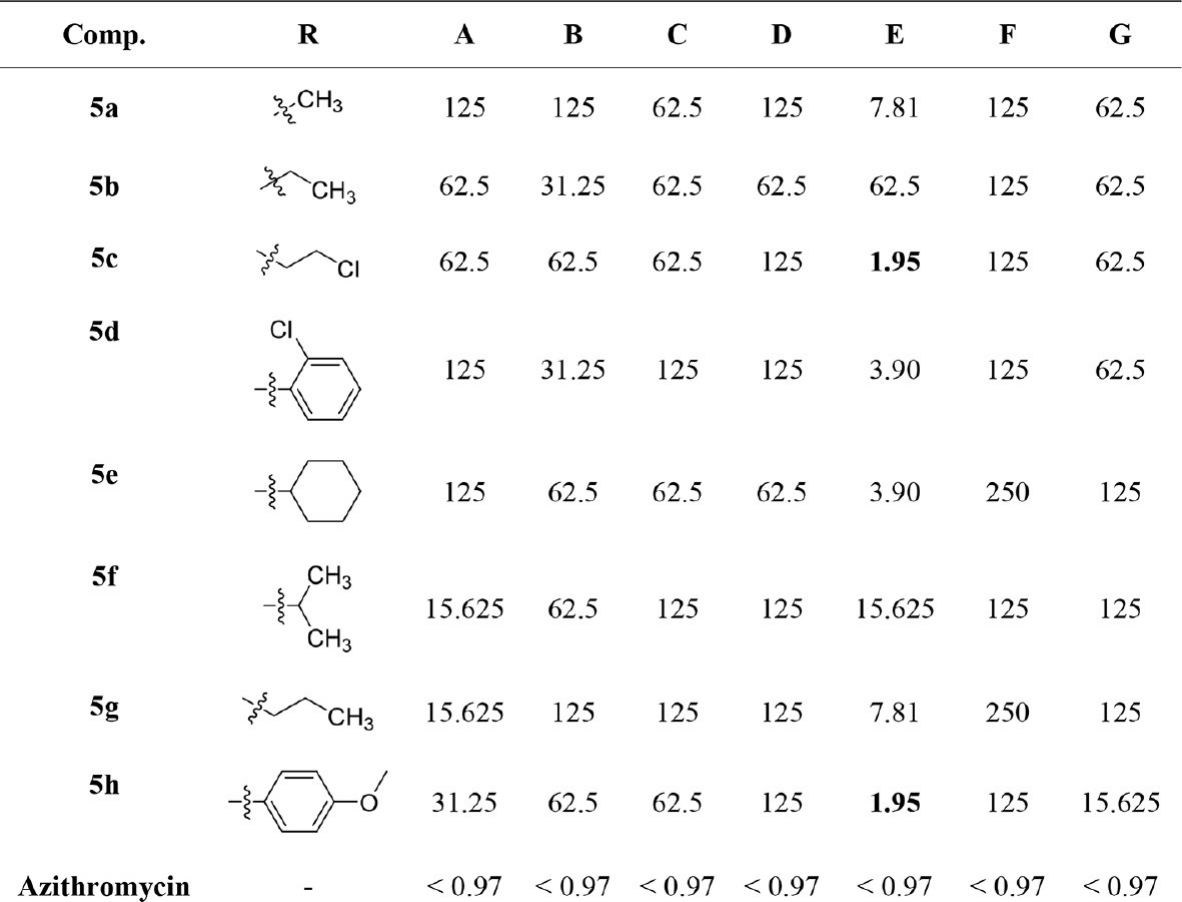
Antifungal Activities of Compounds **5a**–**h** as MIC Values (μg/mL)^a^

a **A**: *C. albicans*. **B**: *C. krusei*., **C**: *C. glabrata*. **D**: *C. parapsilosis*.

The majority of the synthesized compounds effectively suppressed the growth of *C. albicans*, according to the results of the antifungal screening. Compounds **5a**, **5c**, **5d**, **5e**, **5f**, and **5g** were found to be twice as effective as the reference drug voriconazole (MIC = 1.95 μg/mL). In addition, against *C. albicans* compound **5b** had the same effect profile as the reference drug fluconazole (MIC = 7.81 μg/mL). Compound **5f** was the most effective against *C. glabrata*, which was found to be twice as effective as the reference drug voriconazole (MIC = 0.97 μg/mL), whereas compound **5c** showed the same effect profile as the reference drug voriconazole (MIC = 1.95 μg/mL). Compound **5a** showed the same effect profile as the reference drug fluconazole (MIC = 3.90 μg/mL). Compound **5c** was the only compound that was effective against *C. krusei* (MIC = 15.625 μg/mL) and had an effect profile close to that of the reference drug fluconazole (MIC = 7.81 μg/mL). The synthesized compounds have relatively low efficacy as antifungal agents against *C. krusei* and *C. parapsilosis* among the fungus strains.

#### 2.2.2 Antibacterial Activity

In vitro antibacterial screening of the synthesized compounds was assessed against *Escherichia coli* (ATCC 25922), *Serratia marcescens* (ATCC 8100), *Klebsiella pneumoniae* (ATCC 13883), and *Pseudomonas aeruginosa* (ATCC 27853), *Enterococcus faecalis* (ATCC 2942), *Bacillus subtilis* (NRRL NRS 744), *Staphylococcus aureus* (ATCC 29213), and *S. epidermidis* (ATCC 12228). Azithromycin was used as the reference drug for antibacterial activity. The MIC values of the synthesized compounds and the control drug against the bacterial strains are summarized in Table 2. In general, it could be observed that the 2-chloroethyl derivative **5c** and the 4-methoxyphenyl derivative **5h** exhibited potent activity against the Gram-positive bacteria *E. faecalis*, with MIC values of 1.95 μg/mL compared to azithromycin.For these compounds, the electron-withdrawing −Cl group at the 2-position of the ethyl group and the −OCH_3_ group at the 4-position on the phenyl ring, respectively, appeared to increase the antimicrobial activity. Compounds **5d** and **5e** also had notable efficacy (MIC 3.90 μg/mL) against *E. faecalis* ATCC 2942, whereas compounds containing electron-donating CH_3_ groups (**5a**, **5b**, **5f**, and **5g**) had lower antimicrobial activity.

**Table 2:**
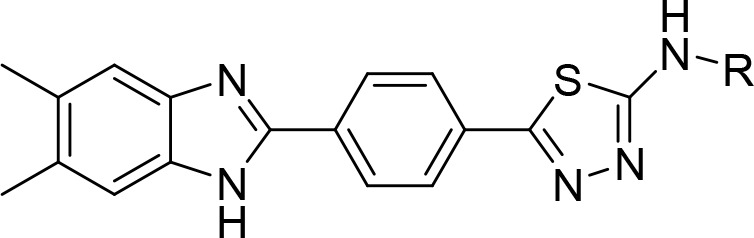

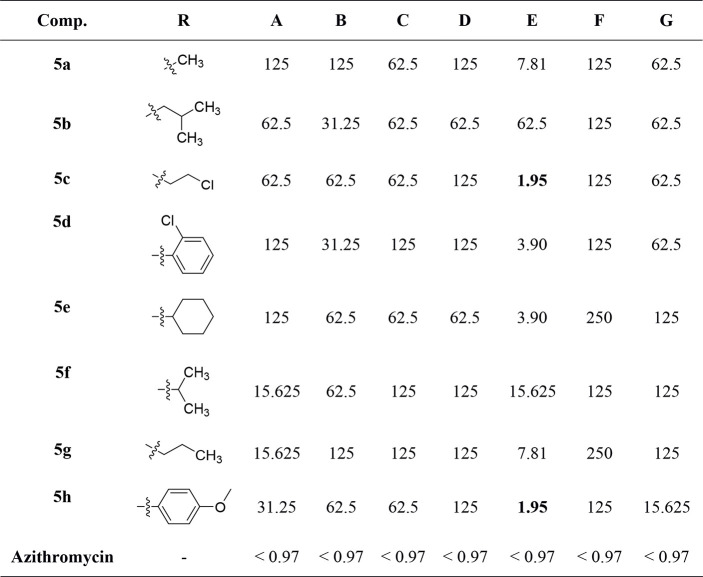
Antibacterial Activities of Compounds **5a**–**h** as MIC Values (μg/mL)^a^

a **A**: *E. coli*. **B**: *S. marcescens*. **C**: *K. pneumoniae*. **D**: *P. aeruginosa*. **E**: *E. faecalis*. **F**: *B. subtilis*. **G**: *S. aureus*. **A**–**D** are Gram-negative bacteria, and **E**–**G** are Gram positive bacteria.

##### Structure–Activity Relationship

**Figure 6:**
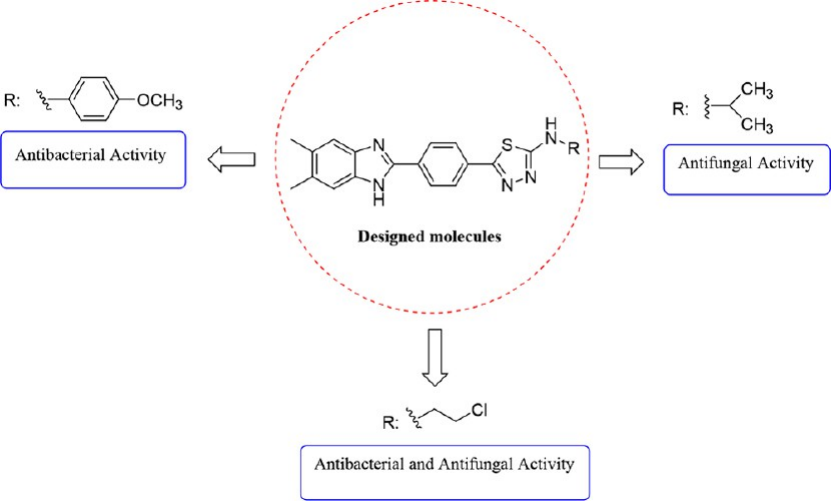
SAR outline for compounds **5a**–**h**.

We discuss the structure–activity relationship (SAR) due to the variations in chemical structures and antimicrobial activity profiles of these substances (Figure 6). The compounds differed in the derivatization of the amine group at the 2-position of the thiadiazole ring. The electron-withdrawing −OCH_3_ at the 4-position of the phenyl substituent (compound **5h**) showed outstanding antibacterial activity with a MIC value of 1.95 μg/mL against *E. faecalis*. The electron-withdrawing −Cl group at the 2-position of the ethyl group in **5c** appears to increase both the antibacterial and antifungal activity. Compounds **5a**–**h** showed relatively similar antifungal activities, which suggests that there are no significant differences in the contributions of different N-substituted moieties to the antifungal activity, making consideration of the SARs very difficult. Looking at the chemical structure of the compounds that showed stronger antifungal activity, one can see that compound **5f** carrying the *N*-isopropyl structure is the most effective compound.

### 2.3 Cytotoxicity Assay

**Figure 7:**
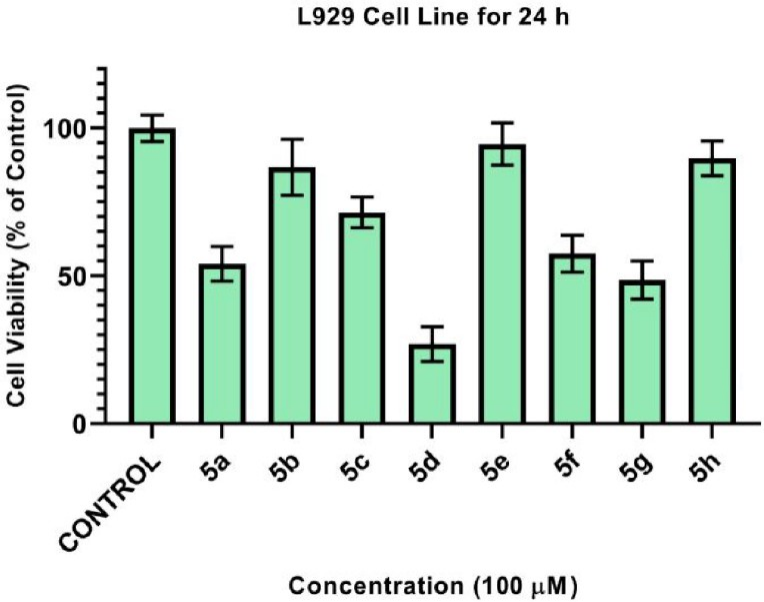
Cell viabilities (%) of the L929 fibroblast cell line against compounds **5a**–**h** for 24 h.

**Figure 8:**
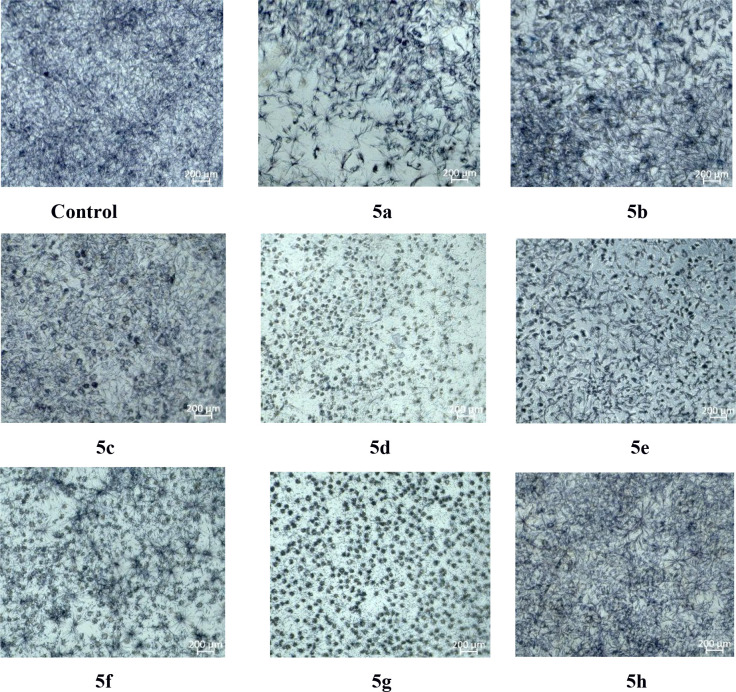
Views of the L929 cell line after administration of the synthesized compounds.

To prove the safety of these compounds to the human body, the cytotoxicity of the compounds to the mouse fibroblast normal cell line (L929) was determined by MTT assay. As shown in Figure 7, compounds **5b**, **5e**, and **5h** were found to have the lowest toxicity. These findings show that the antimicrobial activities of compounds **5e** and **5h** is not due to general toxicity. Views of the L929 cell line after administration of the synthesized compounds are shown in Figure 8.

### 2.4 Molecular Docking Analysis

The interactions of compounds **5a**–**h**, which consist of a substituted thiadiazole derivative bridged to the benzimidazole 2-position by a phenyl group and show good antifungal activity, with the target *Candida* sterol 14-α demethylase (CYP51) were analyzed by the in silico molecular docking method. On the basis of previous studies, benzimidazole derivative compounds show antifungal activity by inhibiting this enzyme.^35−37^ For this reason, CYP51 was identified as the target protein. The azole group of antifungal compounds such as fluconazole and voriconazole is responsible for the interaction with the heme structure in the CYP51 structure, and this interaction is key for inhibition.^38^

**Figure 9:**
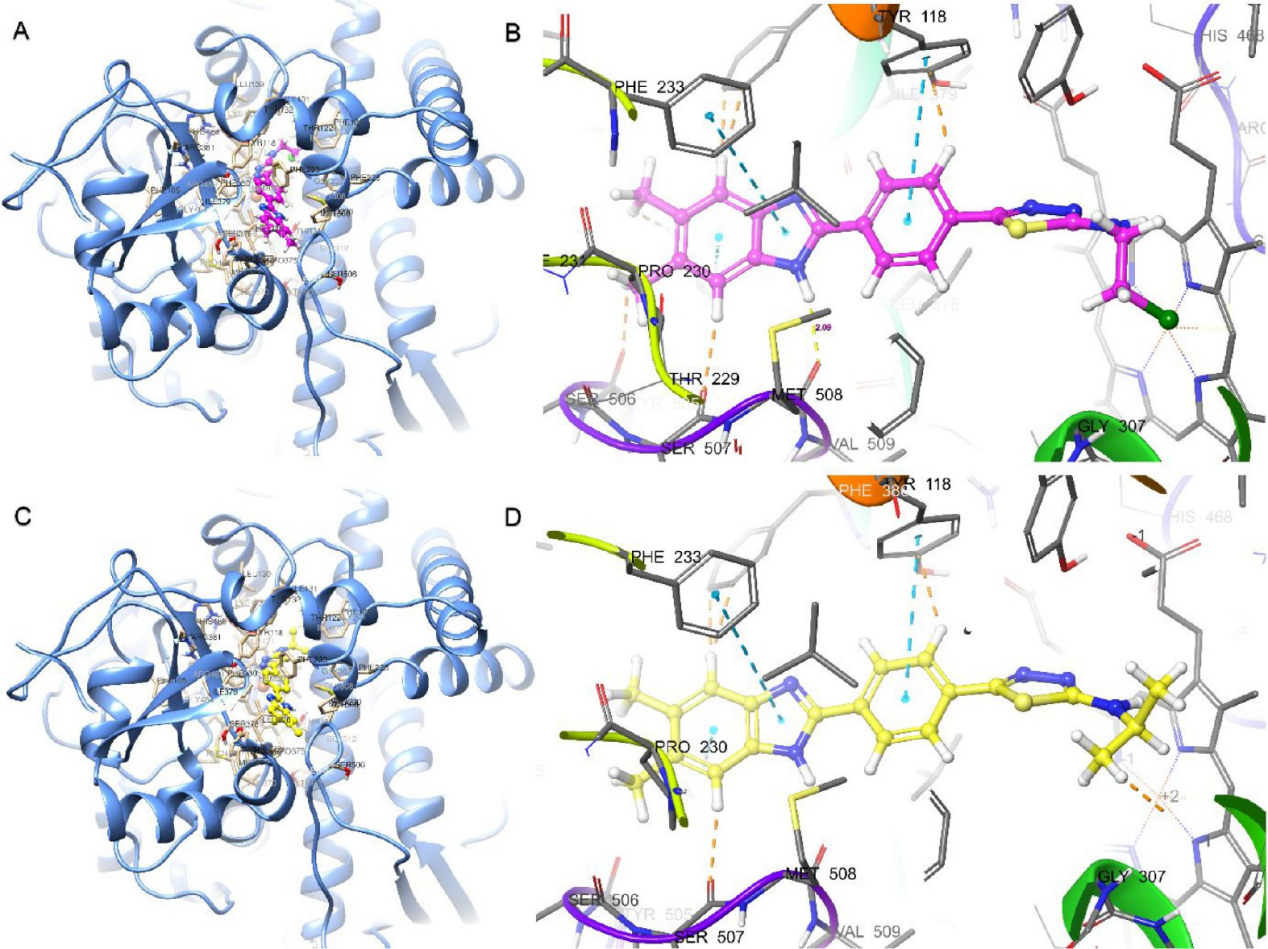
Binding poses and protein–ligand interactions of (A, B) **5c** and (C, D) **5f** with *Candida* sterol 14-α demethylase (CYP51) (PDB ID 5TZ1).

According to molecular docking, the compounds have similar binding poses. As shown in Figure 9, in the benzimidazole–thiadiazole derivative compounds, like azoles, the thiadiazole core is responsible for the interaction with heme. A hydrogen bond is formed between Met508 and the hydrogen at the 1-position of the benzimidazole ring. The molecular docking interaction energies of the compounds are given in Table 3. The most active compound, **5f**, is the compound with the highest docking energy (−10.928 kcal/mol). As with the antifungal activity results, most of the compounds (except **5b** and **5h**) have higher interaction energies than standard fluconazole and voriconazole. As shown in Figure 9B, compound **5c** has a 2.09 Å hydrogen bond between the benzimidazole core H and Met508, a 5.15 Å π–π stacking interaction with heme, and other π–π stacking interactions with Phe233 (5.47 Å), Hie377 (4.46 Å), and Tyr 118 (4.96 Å). In addition, **5c** forms hydrophobic van der Waals interactions with Leu87, Tyr64, Leu376, Ile379, Phe380, Pro230, Phe228, Leu121, Phe126, Ile304, Ile131, Tyr132, and Tyr505. As shown in Figure 9D, as in **5c**, the most active compound **5f** has a 2.10 Å hydrogen bond with Met508 and π–π stacking interactions with heme (5.10 Å), Phe233 (5.44 Å), Hie377 (4.48 Å), and Tyr 118 (4.99 Å). In addition, **5f** gave polar interactions with Hie377, Ser378, Ser506, and Ser507 and hydrophobic interactions with Phe233, Met508, Tyr118, Pro230, Phe233, Leu121, Tyr505, Leu87, Tyr64, Leu376, Ile379, Phe380, Ile304, and Gly303. For compounds **5d** and **5h** carrying the substituted phenyl ring, compound **5e** carrying the cyclohexyl group, and compound **5g** carrying the propyl group, the π–π stacking interactions between the thiadiazole ring and the heme group could not occur. Small groups such as methyl, ethyl, chloroethyl, and isopropyl are more important for interaction. 2D protein–ligand interaction details are given in Figure S1.

**Table 3:** Molecular Docking Binding Energies (kcal/mol) and Some ADME Parameters of Compounds **5a**–**h**, Voriconazole (Vor), and Fluconazole (Flu)

	Glide docking		QikProp ADME				
compd	XP^a^ GScore	MM-GBSA^b^ dG Bind	#stars	QPlogP_o/w_	PHOA^c^	ROF^d^	ROT^e^
**5a**	–8.897	–53.72	0	3.924	100.000	0	1
**5b**	–9.679	–78.24	1	5.219	100.000	1	1
**5c**	–9.975	–70.73	1	4.849	100.000	0	1
**5d**	–8.499	–70.74	1	5.812	100.000	1	1
**5e**	–9.498	–68.40	1	5.536	100.000	1	1
**5f**	–10.928	–60.37	1	4.578	100.000	0	1
**5g**	–10.655	–61.19	1	4.321	100.000	0	1
**5h**	–9.942	–63.29	1	5.372	100.000	1	1
Vor	–6.180	–58.87	0	2.889	100.000	0	0
Flu	–5.235	–50.33	0	0.573	81.840	0	0

a XP: extra precision.

b MM-GBSA: molecular mechanics–generalized Born surface area.

c PHOA: percent human oral absorption.

d ROF: Lipinski’s rule of five.

e ROT: Jorgensen’s rule of three.

### 2.5 Molecular Dynamics Simulations

**Figure 10:**
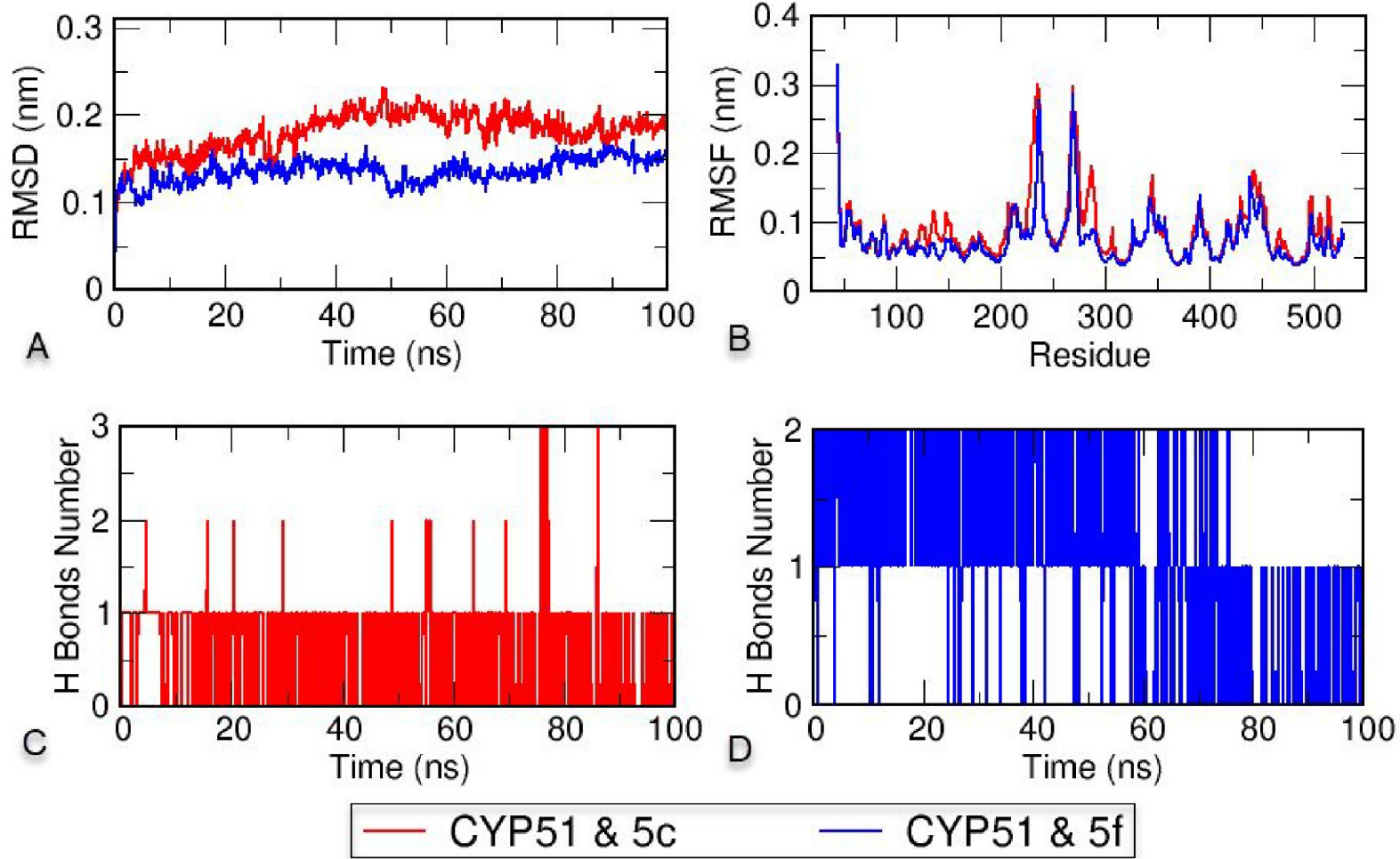
(A) Root-mean-square deviation (RMSD), (B) root-mean-square fluctuation (RMSF), and (C, D) hydrogen bond analysis of the CYP51–**5c** and CYP51–**5f** complexes throughout 100 ns.

To evaluate the stability of the protein–ligand complexes formed by the potential antifungal compounds **5c** and **5f** with *Candida* sterol 14-α demethylase, 100 ns duration MD simulations were performed.^36,39^ Also, the complex of the standard compound voriconazole with CYP51 was simulated for MD validation and compared with the complexes with synthesized compounds **5c** and **5f** (Figure S2). Charmm36m force fields for topology files of CYP51 and compounds **5c** and **5f** were chosen to be compatible with protein–ligand complexes containing the heme structure. The numbers of hydrogen bonds between CYP51 and **5c** and between CYP51 and **5f**, the root-mean-square deviation (RMSD), and the root-mean-square fluctuation (RMSF) were analyzed from the trajectory as time-dependent for 100 ns, and the results are shown in Figure 10. The RMSD, which is used to analyze the deviations in the protein structure, indicates the stability of the protein. As shown in Figure 10A, the CYP51–**5c** complex remained stable at around 0.2 nm, and the CYP51–**5f** complex remained stable below 0.15 nm. RMSF measurements were performed to measure the mobility and flexibility per residue. As shown in Figure 10B, the complex formed by CYP51 and compound **5f** showed less fluctuation than the complex formed by **5c** and made CYP51 more stable. Analyzing the mobility of the protein and ligand system and the hydrogen-bond exchange between the compounds and CYP51 over time is another way of understanding protein–ligand stability. As shown in Figure 10C,D, compound **5c** often formed one hydrogen bond for 100 ns, while compound **5f** formed two hydrogen bonds most of the time. According to the trajectory analysis, stable structures were formed with CYP51 in each compound. However, when the RMSD, RMSF, and hydrogen bond data were examined, compound **5f** formed a more stable protein–ligand complex than voricanazole.

**Figure 11:**
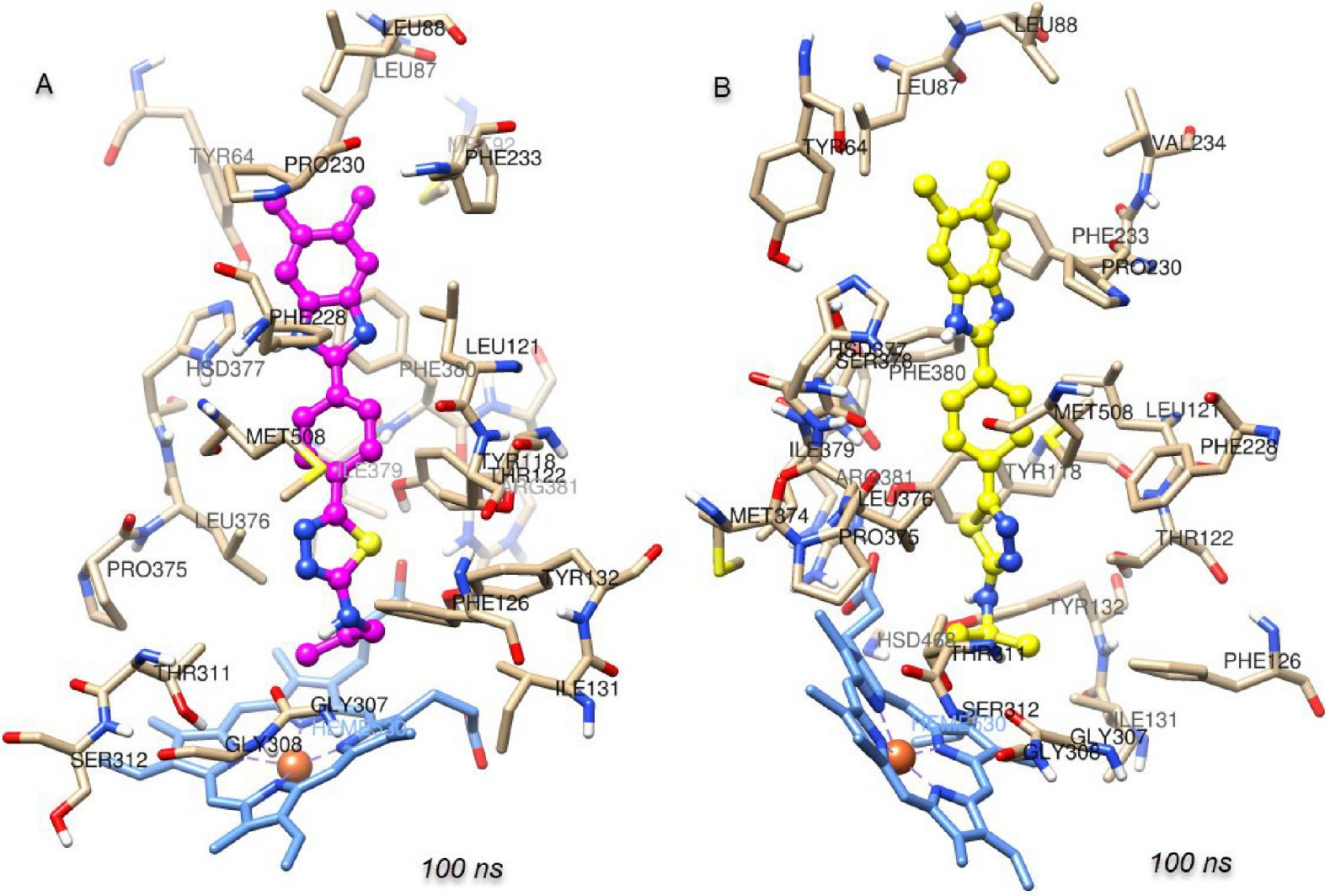
Protein–ligand binding poses of (A) the CYP51–**5c** complex and (B) the CYP51–**5f** complex after 100 ns molecular dynamics simulations.

Animations were created to monitor the changes of compounds **5c** and **5f** over 100 ns (Videos S1 and S2). Both compounds appear to be stable at the CYP51 active site. In addition, protein–ligand binding poses were created to show the positions of the compounds with heme at the end of the MD simulation (Figure 11). Accordingly, although there are changes in some interactions, interactions with heme are preserved, and stability continues.

### 2.6 ADME Estimations

Absorption, distribution, metabolism, and excretion (ADME) estimates of the newly synthesized benzimidazole–thiadiazole hybrids were made using in silico methods with Schrodinger QikProp.^40^ As given in Table 3, the oral absorption of the compounds is full. The lipophilicities of the compounds (in the range of 3.924 to 5.812) are higher than those of fluconazole (0.573) and voriconazole (2.889). The number of stars (#stars), expressing drug-likeness, is obtained from the sum of many parameters and should be in the range of 0–5. The compounds have a #stars value of 1, except for **5a**. According to Lipinski’s rule of five, compounds should have mol_MW < 500, QPlogP_o/w_ < 5, donorHB ≤ 5,and accptHB ≤ 10 and show a deviation of at most 1. According to Jorgensen’s rule of three, compounds should have QPlogS > −5.7, QPPCaco >22 nm/s, and # Primary Metabolites <7 and preferably should show no deviation. Compounds **5a**–**f** comply with Lipinski’s rule of five but deviate by one from Jorgensen’s rule of three. In general, the ADME profiles of the compounds are suitable for limiting rules.

### 2.7 Quantum-Chemical Calculations

**Figure 12:**
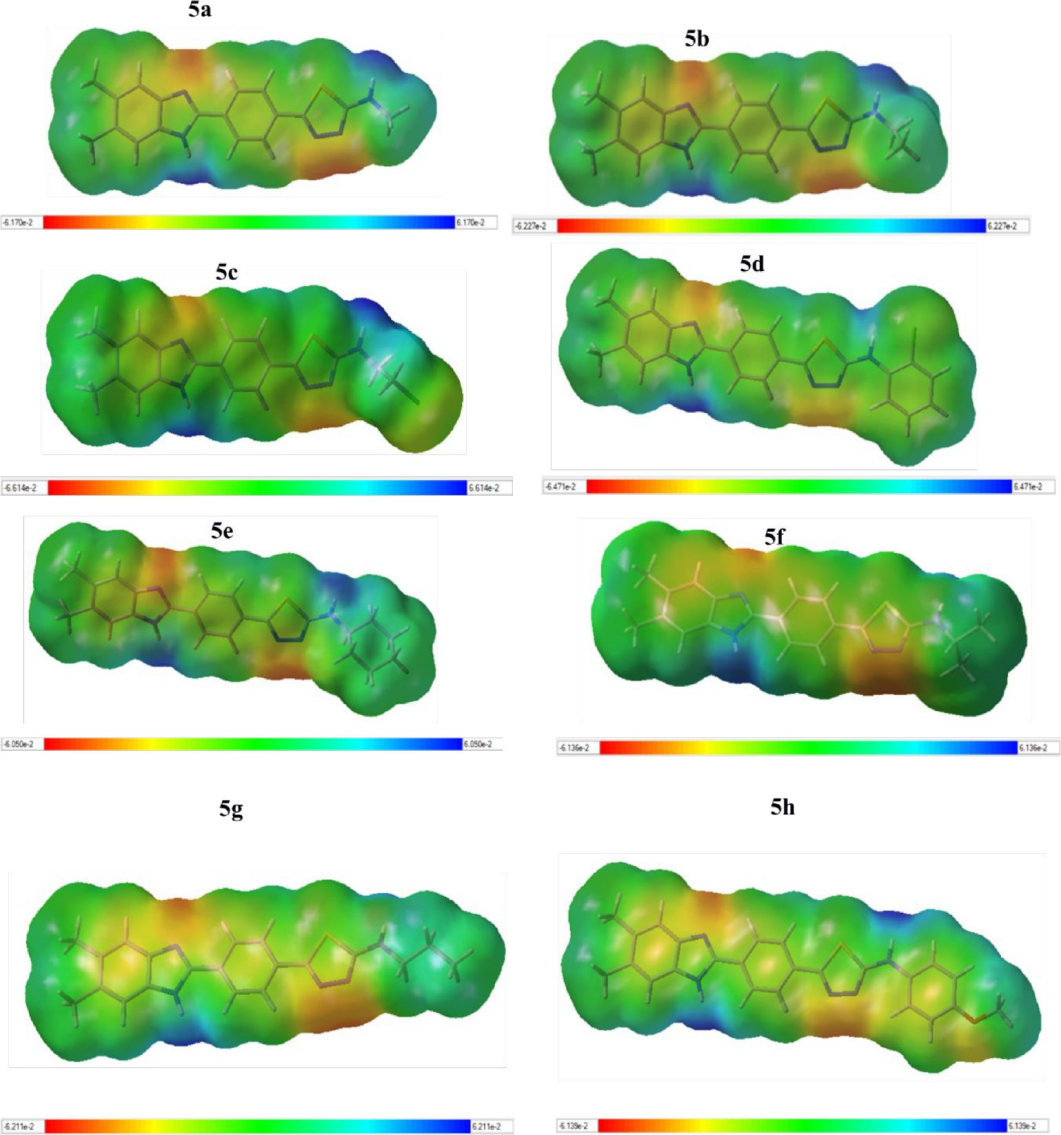
Molecular electrostatic potential (MEP) diagrams for compounds **5a**–**h** at the B3LYP/6-311G(d,p) level. Atom colors: carbon in gray, nitrogen in blue, chlorine in green, oxygen in red, sulfur in yellow, and hydrogen in white. The surfaces plotted by the 0.0004 electrons/Å^3^ contour of the electron density. Color ranges are in a.u.

**Figure 13:**
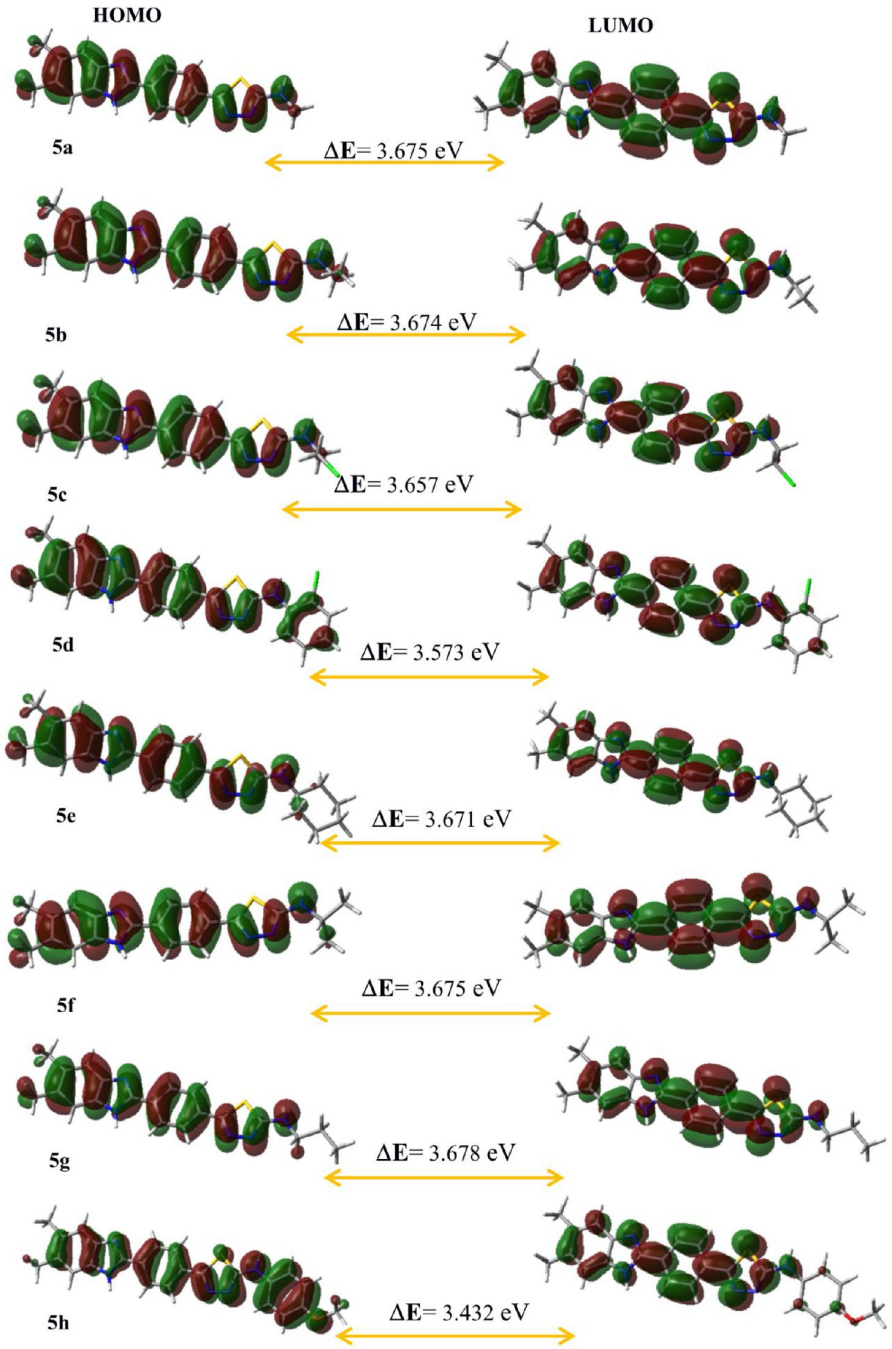
Highest occupied molecular orbital (HOMO) and lowest unoccupied molecular orbital (LUMO) diagrams of current molecules **5a**–**h** calculated at the B3LYP/6-311G(d,p) level. Atom colors: carbon in gray, nitrogen in blue, chlorine in green, oxygen in red, sulfur in yellow, and hydrogen in white.

The optimized geometries of **5a**–**h** and the standard compounds azithromycin, voriconazole, and fluconazole correspond to the true global minima, as no imaginary frequencies were observed in the vibrational frequency investigation. To investigate the electronic characteristics of both these compounds, HOMO and LUMO analyses were carried out. The band gap energy, electron affinity, chemical hardness, ionization potential, electronegativity, chemical potential, chemical softness, and electrophilic index were calculated from the HOMO and LUMO energies. The molecular electrostatic potential (MEP) and HOMO–LUMO diagrams are shown in Figures 12 and 13, respectively. These diagrams for the standard compounds are given in Figures S3 and S4. In addition, the Mulliken atomic charge distributions of **5c**, **5f**, and **5h** are given in Figure S5.

In the MEPs, positive regions indicating low electron density (shown in blue) represent nucleophilic attack, negative areas indicating high electron density (shown in red, orange, or yellow) represent electrophilic attack, and neutral areas are shown in green.^41^ According to the MEP diagrams (Figure 12), while negative regions (red color) representing high electron density in all of the molecules are seen around the N atoms in the benzimidazole and thiadiazole ring, whereas the blue-colored regions with low electron density are formed around the N–H group in the benzimidazole and the N–H group bonded with the thiadiazole ring. This is due to the fact that the nitrogen atoms in the benzimidazole and thiadiazole rings are more basic than those in the N–H group. In Figure 13, it can be seen that the charge distribution in the LUMOs is concentrated on the benzene ring, whereas the HOMO is positioned around the whole of the molecules with the π electron system. The HOMO of π nature (i.e., the benzene ring) is delocalized over the whole C–C bond. The lower energy gap (Δ*E*) means greater chemical reactivity. Understanding the connection between structural stability and global chemical reactivity requires an understanding of global reactivity features. A lower value of the ionization potential (IP) indicates that it has a better electron-donor property.^42^ The calculated values of the electrophilic index (ω) of all molecules belong to the group of “strong electrophiles” (ω > 1.50 eV).^43^ According to Table 4, **5h** is chemically more reactive than the other molecules. The chemical reactivity order is **5h** > **5d** > **5c** > **5e** > **5b** > **5a** = **5f** > **5g** > azithromycin > voriconazole > fluconazole. Moreover, **5f** is chemically more reactive than the standard compounds voriconazole and fluconazole according to the energy gap, as did the antifungal activity results.

**Table 4:** HOMO–LUMO Energies (eV) and Calculated Global Reactivity Parameters^a^ of the Best Stable States of Compounds **5a**–**h** at the B3LYP/6-311G(d,p) Level in the Gas Phase

compd	*E*_LUMO_	*E*_HOMO_	Δ*E*(eV)	IP (eV)	EA (eV)	χ (eV)	η (eV)	*S* (eV)^−1^	μ (eV)	ω (eV)
**5a**	–1.901	–5.576	3.675	5.576	1.900	3.739	1.838	0.272	–3.739	3.803
**5b**	–1.886	–5.560	3.674	5.560	1.886	3.723	1.837	0.272	–3.723	3.773
**5c**	–2.035	–5.692	3.657	5.692	2.035	3.864	1.828	0.273	–3.864	4.083
**5d**	–2.120	–5.693	3.573	5.693	2.120	3.907	1.787	0.280	–3.907	4.272
**5e**	–1.857	–5.528	3.671	5.528	1.857	3.693	1.836	0.272	–3.693	3.715
**5f**	–1.878	–5.553	3.675	5.553	1.878	3.715	1.838	0.272	–3.715	3.756
**5g**	–1.882	–5.560	3.678	5.560	1.882	3.721	1.839	0.272	–3.721	3.764
**5h**	–1.981	–5.413	3.432	5.413	1.981	3.697	1.716	0.291	–3.697	3.982
Vor^b^	–1.727	–7.070	5.343	7.070	1.727	4.398	2.671	0.187	–4.398	3.621
Flu^c^	–1.039	–7.238	6.199	7.238	1.039	4.139	3.099	0.161	–4.139	2,763
Azm^d^	–0.280	–5.052	4.772	5.052	0.280	2.666	2.386	0.209	–2.666	1.490

a Δ*E* is the band gap (*E*_LUMO_ – *E*_HOMO_). IP is the ionization potential (=–*E*_HOMO_). EA is the electron affinity (=–*E*_LUMO_). χ is the electronegativity (=(IP + EA)/2). η is the chemical hardness (=(IP – EA)/2). *S* is the chemical softness (=1/2η). μ is the chemical potential (=–(IP + EA)/2). ω is the electrophilic index (=μ^2^/2η).

b Vor = voriconazole.

c Flu = fluconazole.

d Azm = azithromycin.

## 3 Conclusion

We synthesized novel benzimidazole–1,3,4-thiadiazole hybrid compounds, and their chemical structures were elucidated by ^1^H and ^13^C NMR, HRMS, and elemental analysis. The antimicrobial activities of the new compounds were tested against *E. coli*, *S. marcescens*, *K. pneumoniae*, *P. aeruginosa*, *E. faecalis*, *B. subtilis*, *S. aureus*, and four *Candida* species in vitro. The novel derivatives were more active against Gram-negative bacteria and *Candida* species than Gram-positive bacteria. Compounds **5c** and **5h** had a substantial antimicrobial effect against *E. faecalis*, while the other derivatives demonstrated relatively moderate activity. Many of the compounds appear to have significant activity against *C. albicans* with a MIC value of 1.95 μg/mL. Additionally, docking studies were performed on the most promising compounds **5c** and **5f** in the active site of 14-α demethylase. According to MD trajectory analysis, compound **5f** was more potent than **5c**, in accordance with the antifungal activities and docking scores. In addition, **5f** is chemically more reactive than the standard compounds (voriconazole and fluconazole) according to the energy gaps obtained from HOMO–LUMO analyses and the biological activity results against *C. albicans* and *C. glabrata*. ADME analysis of the synthesized compounds was performed. The hydrogen at the 1-position of the benzimidazole ring in all of the molecules are responsible for nucleophilic attack according to the MEP diagrams. This state supports the intermolecular hydrogen bond between Met508 and the hydrogen at the 1-position of the benzimidazole ring in accordance with molecular docking analysis.

## 4 Materials and Methods

### 4.1 Synthesis

All of the chemicals used in the synthetic process were bought from Merck (Darmstadt, Germany) or Sigma-Aldrich (St. Louis, MO, USA). The MP90 digital melting point instrument (Mettler Toledo, OH, USA) was used to determine the uncorrected melting points of the compounds that were obtained. A Bruker digital FT NMR spectrometer (Bruker Bioscience, Billerica, MA, USA) was used to record the ^1^H and ^13^C NMR spectra of the produced compounds in DMSO-*d*_6_. Splitting patterns in the NMR spectra were designated as follows: s, singlet; d, doublet; t, triplet; m, multiplet. Coupling constants (*J*) are reported in hertz. [M + 1] peaks were determined using a Shimadzu LC/MSMS system (Shimadzu, Tokyo, Japan). Using silica gel 60 F_254_ TLC plates (Merck), thin-layer chromatography (TLC) was used to monitor each reaction.

#### 4.1.1 Methyl 4-Formylbenzoate Sodium Metabisulfite Salt Derivative (**1**)

Ethanol was used to dissolve methyl 4-formylbenzoate (5 g, 0.03 mol), and ethanol-dissolved sodium metabisulfite (6.84 g, 0.036 mol) was added dropwise to the benzaldehyde solution. The reaction components were mixed for 1 h at room temperature once the addition process was finished, and the reaction solution was filtered to remove the precipitated product.

#### 4.1.2 Methyl 4-(5,6-Dimethyl-1*H*-benzimidazol-2-yl)benzoate (**2**)

5,6-Dimethylbenzene-1,2-diamine (0.022 mol) was dissolved in DMF, and compound **1** (7.09 g, 0.026 mol) was then added. By adding the reaction contents to iced water at the end, the result was precipitated. From ethanol, the precipitated product was removed and crystallized.

#### 4.1.3 4-(5,6-Dimethyl-1*H*-benzimidazol-2-yl)benzene-1-carbohydrazide (**3**)

The same vial was filled with compound **2** (0.018 mol), an excess of hydrazine hydrate (5 mL), and ethanol (15 mL). For 12 h, the mixture was refluxed. The liquid was placed into iced water when the reaction was finished, and the end result was filtered.

#### 4.1.4 N-Substituted 2-(4-(5,6-Dimethyl-1*H*-benzimidazol-2-yl)benzoyl)hydrazine-1-carbothioamides (**4a**–**h**)

Compound **3** (1 mmol) was dissolved in 10 mL of ethanol, and the appropriate isothiocyanate (1.1 mmol) was added. The reaction mixture was refluxed for 3 h, and the precipitated product was filtered off.

#### 4.1.5 N-Substituted 5-(4-(5,6-Dimethyl-1*H*-benzimidazol-2-yl)phenyl)-1,3,4-thiadiazole-2-amines (**5a**–**h**)

The appropriate thiosemicarbazide derivative **4** was stirred in 10 mL of H_2_SO_4_ in an ice bath. The mixture was stirred at room temperature for 10 min and then poured into ice water, and the pH was adjusted to 8 with aqueous ammonia. The product was washed with water and crystallized from ethanol. ^1^H NMR, ^13^C NMR, and HRMS spectra of compounds **5a**–**h** are given in Figures S6–S27.

##### *N*-Methyl-5-(4-(5,6-dimethyl-1*H*-benzimidazol-2-yl)phenyl)-1,3,4-thiadiazole-2-amine (**5a**)

Yield: 68%. Mp: 311.8 °C. ^1^H NMR (300 MHz, DMSO-*d*_6_): δ = 2.35 (6H, s, −CH_3_), 2.95 (3H, s, −CH_3_), 7.47–7.48 (2H, m, aromatic CH), 7.95–8.01 (2H, m, aromatic CH), 8.23–8.26 (1H, m, aromatic CH), 8.31–8.33 (1H, m, aromatic CH). ^13^C NMR (75 MHz, DMSO-*d*_6_): δ = 20.52, 35.85, 125.66, 126.04, 127.32, 127.48, 128.23, 128.92, 129.43, 131.21, 133.17, 145.70, 164.40, 168.92. HRMS (*m*/*z*): [M + H]^+^ calcd for C_18_H_17_N_5_S 336.1277, found 336.1271. Anal. Calcd for C_18_H_17_N_5_S: C, 64.45; H, 5.11; N, 20.88. Found: C, 64.62; H, 5.10; N, 20.91.

##### *N*-Ethyl-5-(4-(5,6-dimethyl-1*H*-benzimidazol-2-yl)phenyl)-1,3,4-thiadiazole-2-amine (**5b**)

Yield: 69%. Mp: 299.2 °C. ^1^H NMR (300 MHz, DMSO-*d*_6_): δ = 1.22 (3H, t, *J* = 7.17 Hz, −CH_3_), 2.40 (6H, s, −CH_3_), 3.37 (2H, q, *J* = 6.99 Hz, CH_2_), 7.62–7.63 (2H, m, aromatic C–H), 8.05–8.08 (2H, m, aromatic CH), 8.23–8.27 (2H, m, aromatic CH). ^13^C NMR (75 MHz, DMSO-*d*_6_): δ = 20.47, 25.14, 38.21, 114.06, 114.57, 118.17, 124.52, 127.51, 128.88, 130.74, 134.92, 135.25, 136.31, 147.28, 154.58, 169.59. HRMS (*m*/*z*): [M + H]^+^ calcd for C_19_H_19_N_5_S 350.1434, found 350.1432. Anal. Calcd for C_19_H_19_N_5_S: C, 65.30; H, 5.48; N, 20.04. Found: C, 65.42; H, 5.46; N, 20.08.

##### *N*-(2-Chloroethyl)-5-(4-(5,6-dimethyl-1*H*-benzimidazol-2-yl)phenyl)-1,3,4-thiadiazole-2-amine (**5c**)

Yield: 66%. Mp: 202.4 °C. ^1^H NMR (300 MHz, DMSO-*d*_6_): δ = 2.32 (6H, s, −CH_3_), 3.37 (4H, s, −CH_2_), 7.39–7.41 (2H, m, aromatic CH), 7.95–7.97 (1H, m, aromatic CH), 8.06–8.09 (1H, m, aromatic C–H), 8.23–8.28 (2H, m, aromatic CH). ^13^C NMR (75 MHz, DMSO-*d*_6_): δ = 21.40, 45.20, 55.69, 112.22, 113.98, 119.38, 124.27, 125.93, 126.55, 129.29, 134.03, 135.70, 137.46, 142.66, 147.75, 160.11. Anal. Calcd for C_19_H_18_N_5_SCl: C, 59.44; H, 4.73; N, 18.24. Found: C, 59.56; H, 4.72; N, 18.28.

##### *N*-(2-Chlorophenyl)-5-(4-(5,6-dimethyl-1*H*-benzimidazol-2-yl)phenyl)-1,3,4-thiadiazole-2-amine (**5d**)

Yield: 70%. Mp: 262.8 °C. ^1^H NMR (300 MHz, DMSO-*d*_6_): δ = 2.44 (6H, s, −CH_3_), 7.06–7.09 (2H, m, aromatic CH), 7.38–7.42 (2H, m, aromatic CH), 7.51–7.54 (2H, m, aromatic CH), 8.02–8.04 (2H, m, aromatic CH), 8.27–8.31 (2H, m, aromatic CH). ^13^C NMR (75 MHz, DMSO-*d*_6_): δ = 22.65, 123.59, 125.76, 126.47, 126.60, 127.32, 128.66, 129.57, 131.38, 131.75, 132.36, 132.57, 133.14, 134.69, 136.10, 137.79, 139.23, 139.44, 150.20, 165.58. HRMS (*m*/*z*): [M + H]^+^ calcd for C_23_H_18_N_5_SCl 432.1044, found 432.1059. Anal. Calcd for C_23_H_18_N_5_SCl: C, 63.95; H, 4.20; N, 16.21. Found: C, 64.17; H, 4.19; N, 16.25.

##### *N*-Cyclohexyl-5-(4-(5,6-dimethyl-1*H*-benzimidazol-2-yl)phenyl)-1,3,4-thiadiazole-2-amine (**5e**)

Yield: 73%. Mp: 241.5 °C. ^1^H NMR (300 MHz, DMSO-*d*_6_): δ = 1.17–1.36 (5H, m, cyclohexyl CH), 1.56–1.71 (3H, m, cyclohexyl CH), 1.99–2.02 (2H, m, cyclohexyl CH), 2.38 (6H, s, −CH_3_), 3.58 (1H, s, cyclohexyl CH), 7.54 (2H, s, aromatic CH), 8.00 (2H, d, *J* = 7.80 Hz, aromatic CH), 8.22 (2H, d, *J* = 7.92 Hz, aromatic CH). ^13^C NMR (75 MHz, DMSO-*d*_6_): δ = 20.45, 21.29, 23.01, 24.76, 25.66, 32.49, 54.35, 114.46, 115.18, 126.02, 126.25, 127.36, 128.39, 129.43, 132.96, 134.23, 134.91, 148.11, 154.65, 168.71. HRMS (*m*/*z*): [M + H]^+^ calcd for C_23_H_25_N_5_S 404.1903, found 404.1919. Anal. Calcd for C_23_H_25_N_5_S: C, 68.46; H, 6.24; N, 17.35. Found: C, 68.70; H, 6.22; N, 17.41.

##### *N*-Isopropyl-5-(4-(5,6-dimethyl-1*H*-benzimidazol-2-yl)phenyl)-1,3,4-thiadiazole-2-amine (**5f**)

Yield: 73%. Mp: 292.8 °C. ^1^H NMR (300 MHz, DMSO-*d*_6_): δ = 0.93 (2H, d, *J* = 6.69 Hz, CH_3_), 2.40 (6H, s, −CH_3_), 3.18–3.20 (1H, m, −CH), 7.64 (2H, s, aromatic CH), 8.07 (2H, d, *J* = 8.88 Hz, aromatic CH), 8.27 (2H, d, *J* = 8.67 Hz, aromatic CH). ^13^C NMR (75 MHz, DMSO-*d*_6_): δ = 19.43, 21.26, 23.59, 107.23, 109.72, 115.20, 115.99, 124.06, 124.48, 126.48, 127.81, 130.74, 134.81, 136.81, 136.94, 154.09. Anal. Calcd for C_20_H_21_N_5_S: C, 66.09; H, 5.82; N, 19.27. Found: C, 66.23; H, 5.80; N, 19.30.

##### *N*-Propyl-5-(4-(5,6-dimethyl-1*H*-benzimidazol-2-yl)phenyl)-1,3,4-thiadiazole-2-amine (**5g**)

Yield: 68%. Mp: 148.4 °C. ^1^H NMR (300 MHz, DMSO-*d*_6_): δ = 0.94 (3H, t, *J* = 7.41 Hz, −CH_3_), 1.60 (2H, m, −CH_2_), 2.33 (6H, s, CH_3_), 3.22–3.24 (2H, m, CH_2_), 7.38 (2H, s, aromatic CH), 7.90 (2H, d, *J* = 8.61 Hz, aromatic CH), 8.21 (2H, d, *J* = 8.25 Hz, aromatic CH). ^13^C NMR (75 MHz, DMSO-*d*_6_): δ = 11.12, 20.99, 25.87, 29.09, 112.84, 115.33, 123.54, 126.35, 127.40, 129.67, 130.92, 134.45, 135.59, 141.72, 148.58, 152.11, 169.72. HRMS (*m*/*z*): [M + H]^+^ calcd for C_20_H_21_N_5_S 364.1590, found 364.1601. Anal. Calcd for C_20_H_21_N_5_S: C, 66.09; H, 5.82; N, 19.27. Found: C, 66.18; H, 5.81; N, 19.31.

##### *N*-(4-Methoxyphenyl)-5-(4-(5,6-dimethyl-1*H*-benzimidazol-2-yl)phenyl)-1,3,4-thiadiazole-2-amine (**5h**)

Yield: 74%. Mp: 319.9 °C. ^1^H NMR (300 MHz, DMSO-*d*_6_): δ = 2.33 (6H, s, −CH_3_), 3.75 (3H, s, −OCH_3_), 6.99 (2H, d, *J* = 8.85 Hz, aromatic CH), 7.12 (2H, br s, aromatic C–H), 7.76 (1H, dd, *J*_1_ = 2.88 Hz, *J*_2_ = 8.76 Hz, aromatic CH), 7.86–7.87 (1H, m, aromatic CH), 7.99 (2H, d, *J* = 8.52 Hz, aromatic CH), 8.24 (2H, d, *J* = 8.52 Hz, aromatic CH). ^13^C NMR (75 MHz, DMSO-*d*_6_): δ = 20.52, 56.23, 113.13, 115.02, 119.21, 119.62, 120.24, 122.78, 127.30, 127.52, 129.16, 131.50, 132.00, 133.06, 136.75, 149.92, 152.31, 156.75, 165.42. HRMS (*m*/*z*): [M + H]^+^ calcd for C_24_H_21_N_5_OS 428.1540, found 428.1540. Anal. Calcd for C_24_H_21_N_5_OS: C, 67.43; H, 4.95; N, 16.38. Found: C, 67.54; H, 4.94; N, 16.40.

### 4.2 Antimicrobial Assay

The minimum inhibitory concentration (MIC) tests using 96-well microplates were performed by the microdilution standard methods CLSI M07-A9 (2012)^44^ and NCCLS M27-A2 (2002)^45^ for bacteria and yeasts, respectively. The test microorganisms in the antibacterial assays were *E. coli* (ATCC 25922), *S. marcescens* (ATCC 8100), *K. pneumonia* (ATCC 13883), *P. aeruginosa* (ATCC 27853), *E. faecalis* (ATCC 2942), *B. subtilis* (NRRL NRS 744), *S. aureus* (ATCC 29213), *S. epidermidis* (ATCC 12228), and in the antifungal assays *C. albicans* ATCC 24433, *C. glabrata* (ATCC 90030), *C. krusei* ATCC 6258, and *C. parapsilosis* (ATCC 22019) were used. MHA and MHB media for bacteria and SDA and SDB media were used for the growth of test microorganisms used in the antimicrobial experiments. The wells were prepared to contain a volume of 100 μL in the final case. Using 1000 ppm stock solutions, each compound concentration in the first well was prepared to be 250 μg/mL. Serial dilutions were made in a 1:2 ratio, and the concentrations of the next wells were adjusted to 125, 62.5, 31.25, 15.625, 7.81, 3.90, 1.95, and 0.97 μg/mL, respectively. The microbial suspension adjusted to a 0.5 McFarland standard was inoculated into wells. The bacteria and yeasts were incubated for 24 h at 37 °C and 48 h at 35 °C, respectively. The same procedures were applied to azithromycin for bacteria as the positive control. Voriconazole and fluconazole were used as the positive controls for yeast cells. Negative controls were pure liquids devoid of any microbes. Turbidity that appeared after 24–48 h was used to compare both positive and negative outcomes to those in the control wells. The lowest concentration at which each microbe showed no discernible growth was designated as the MIC. The turbidity showing microbial growth after incubation was measured as the optical density at 600 nm with a multiwell plate reader (Multiskan FC, Thermo Scientific, Waltham, MA, USA). All of the chemicals used in the experiments were purchased from Merck. All of the antimicrobial studies were carried out in triplicate with aseptic conditions.

### 4.3 Cytotoxicity Assay

#### 4.3.1 Cell Culture

The fibroblast cell line L929 was obtained from the American Type Culture Collection and cultured in Dulbecco’s modified Eagle’s medium (DMEM) (Thermo Fisher Scientific) supplemented with 10% fetal bovine serum (FBS) (Sigma-Aldrich), 1% l-glutamine (Sigma-Aldrich), and 1% penicillin/streptomycin (Sigma-Aldrich). The cultivated cells were kept at a temperature of 37 °C in a humid environment that contained 5% CO_2_. Since the final DMSO concentration did not go over 0.5%, all of the recently produced compounds were dissolved in DMSO, and stock solutions were diluted with DMEM.

#### 4.3.2 Cell Viability Assay

The MTT assay was used to examine how **5a**–**h** affected the viability of the L929 cell line. The cells were treated with 100 M concentrations of each after being plated at a density of 1 × 10^4^ cells per well for 24 h. Untreated cells were used as control. After incubation, 20 μL of MTT solution (5 mg/mL in PBS, Sigma) was added to the cells, and they were then incubated at 37 °C for 3 h to allow the metabolically active cells to convert the MTT dye into formazan crystals. The formazan crystals were dissolved in DMSO (Sigma). Utilizing a microplate reader (Thermo, Germany) to measure the absorbance at 540 nm, the decrease of MTT was measured. Data are reported as mean ± standard deviation.

### 4.4 Molecular Modeling

#### 4.4.1 Molecular Docking

Molecular docking studies were carried out using the Maestro molecular modeling environment of the Schrödinger version 2021–2. Since the mechanism of action of benzimidazole derivatives is via 14-α demethylase (CYP51)^46^ of *Candida*, the CYP51 structure was downloaded from the Protein Data Bank (PDB) with PDB ID 5TZ1 and prepared using the OPLS4 force field with Protein Preparation Wizard. The structures of the compounds were drawn with ChemDraw Professional 16 version, and 3D minimized structures obtained from DFT studies with the B3LYP/6-311G(d,p) basis set. Active-site coordinates were determined as (*x*, *y*, *z*) = (70.610, 66.284, 4.177) based on the cocrystal ligand VT1 in the 5TZ1 structure and prepared as 20 Å × 20 Å × 20 Å by Receptor Grid Preparation. Molecular docking was performed with Glide XP ligand docking.^47^ For validation of the docking study, cocrystal VT1 redocking was performed, and the RMSD between the natural pose and docking pose was measured as 0.3865 Å. Visualization and detailed analysis of protein–ligand interactions were performed with UCSF Chimera version 1.16 and the Maestro Ligand Interaction module.

#### 4.4.2 Molecular Dynamics Simulations

MD simulations were performed with Gromacs version 2020.4 as in our previous studies.^48−50^ Input files were created via CHARMM-GUI server solution builder (https://charmm-gui.org/).^51^ The topology file of CYP51 protein, heme and compounds **5c** and **5f** were prepared using the Charmm36m force field.^52^ The TIP3 water model was used for solvation and neutralized with 0.15 M KCl. The rectangular box system consisting of protein, heme, compounds, water, and ions was minimized in 5000 steps. It was equilibrated with the *NVT*/*NPT* ensemble at 1 atm pressure and 300 K temperature for 300 ps. The 100 ns MD simulations were run for 1000 frames to 2 fs. RMSD, RMSF, and hydrogen-bond analyses were performed with gmx scripts from the trajectory. MD graphics were created with QtGrace tool version 0.2.6 and animation videos with UCSF Chimera version 1.16.

#### 4.4.3 ADME Estimations

The QikProp module of Schrodinger version 2021–2 was used to do prediction studies of in silico ADME features of compounds.^38^

#### 4.4.4 Quantum-Chemical Computations

In the structure–activity relationship, it is essential to determine the accurate structure. For this purpose, the eight newly synthesized compounds were modeled with density functional theory. The DFT calculations were performed using the Gaussian 09 package^53^ at the B3LYP/6-311G(d,p) level of theory to find steady states of the models. GaussView 5.0 was used to visualize the optimized geometries.^30^

## Supporting Information Available

^1^H NMR, ^13^C NMR, and HRMS spectra, molecular docking binding poses, MD trajectory analysis, HOMO and LUMO plots, MEP diagrams, and Mulliken atomic charge distributions (PDF)
MD animation videos (ZIP)
