# Supplementary material for: Synthesis, Molecular Docking, Dynamics, Quantum-Chemical Computation, and Antimicrobial Activity Studies of Some New Benzimidazole–Thiadiazole Hybrids
Source: ACS Omega. 2022 Dec 9;7(50):47015–30. doi: 10.1021/acsomega.2c06142 (PMC9773947; doi:10.1021/acsomega.2c06142)
Supplement: Supplementary file 1 — ao2c06142_si_001.pdf [file ao2c06142_si_001.pdf]

Synthesis, Molecular Docking, Dynamics, Quantum-Chemical Computation, and Antimicrobial Activity Studies of Some New Benzimidazole–Thiadiazole Hybrids

Ismail Celik*[1], Ulviye Acar Çevik[2], Arzu Karayel[3], Ayşen Işık[4], Uğur Kayış[5], Ülküye Dudu Gül[6], Hayrani Eren Bostancı[7], Süheyl Furkan Konca[8], Yusuf Özkay[2], Zafer Asım Kaplancıklı[2]

[1] Department of Pharmaceutical Chemistry, Faculty of Pharmacy, Erciyes University, Kayseri 38039, Turkey.

[2] Department of Pharmaceutical Chemistry, Faculty of Pharmacy, Anadolu University, Eskişehir 26470, Turkey.

[3] Department of Physics, Faculty of Arts and Science, Hitit University, 19030 Çorum, Turkey.

[4] Department of Biochemistry, Faculty of Science, Selçuk University, 42250 Konya, Turkey.

[5] Pazaryeri Vocational School, Program of Pharmacy Services, Bilecik Şey Edebali University, 11230 Bilecik, Turkey.

[6] Department of Bioengineering, Faculty of Engineering, Bilecik Seyh Edebali University, 11230 Bilecik, Turkey.

[7] Department of Pharmaceutical Basic Sciences, Cumhuriyet University, Faculty of Pharmacy, 58140 Sivas, Turkey

[8] Department of Pharmaceutical Biotechnology, Faculty of Pharmacy, Erciyes University, 38039 Kayseri, Turkey.

*Corresponding Author. E-mail: ismailcelik@erciyes.edu.tr; Tel. +90-222-335-0580/3775

Address: Erciyes University, Faculty of Pharmacy, Department of Pharmaceutical Chemistry, 38039, Kayseri, Turkey.

Sr. No.	Table of contents	Page No.
1	Molecular docking binding poses of compounds **5a-h,** voriconazole and fluconazole with CYP51	S2
2	Molecular dynamics trajectory analysis of of CYP51 & voriconazole complex	S3
3	HOMO and LUMO plots of azithromycin, voriconazole and fluconazole	S4
4	MEP diagrams of standard compounds azithromycin, voriconazole and fluconazole	S5
5	The mulliken atomic charge distribution of **5c**, **5f** and **5h**	S6
6	^{1}H-NMR, ^{13}C-NMR and HRMS spectrum of compounds **5a-h**	S7-20

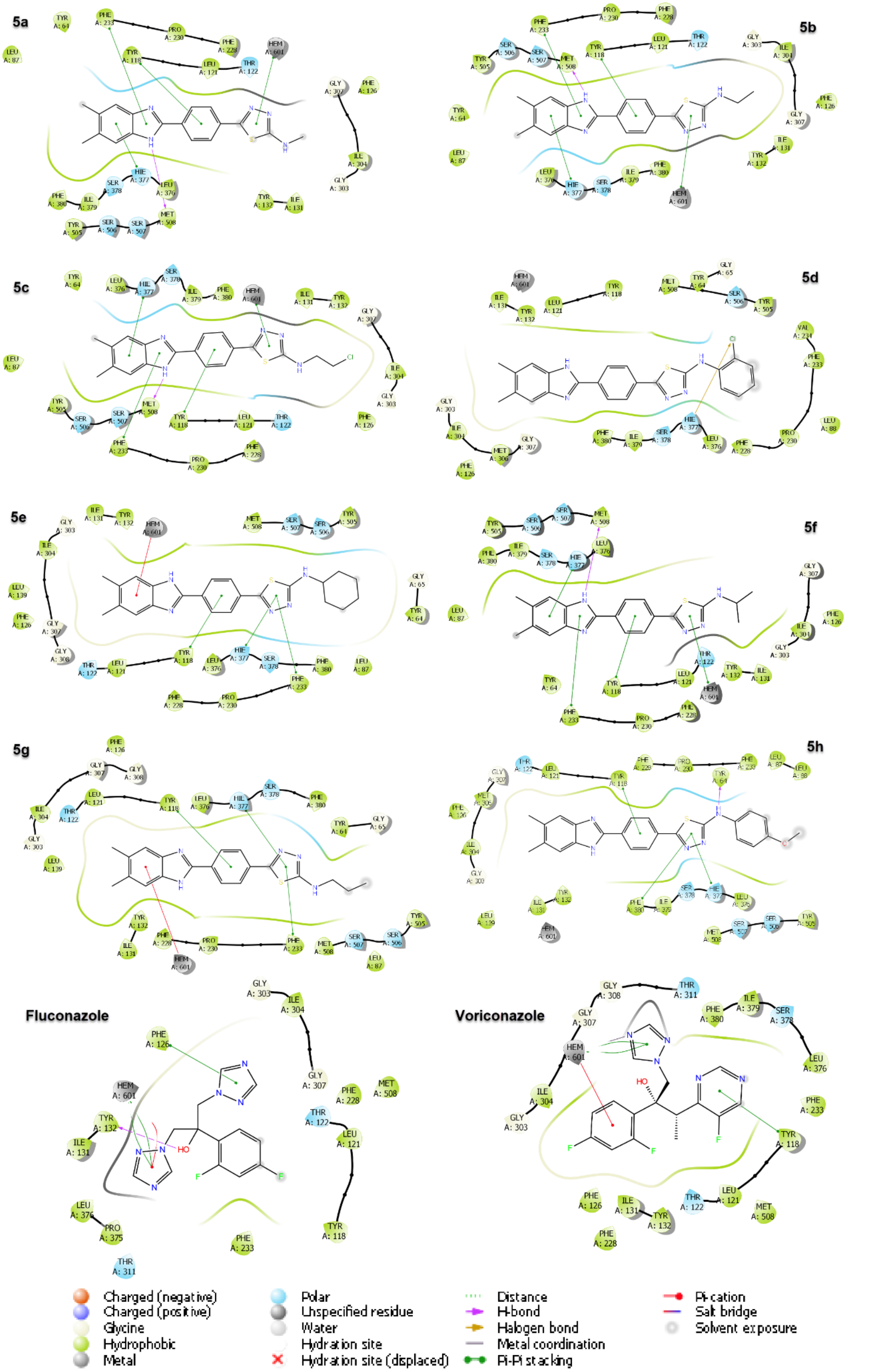

Figure S1. 2D interaction diagrams of compounds **5a-h**, fluconazole and voriconazole with Candida's 14-alpha demethylase enzyme (PDB ID: 5TZ1)

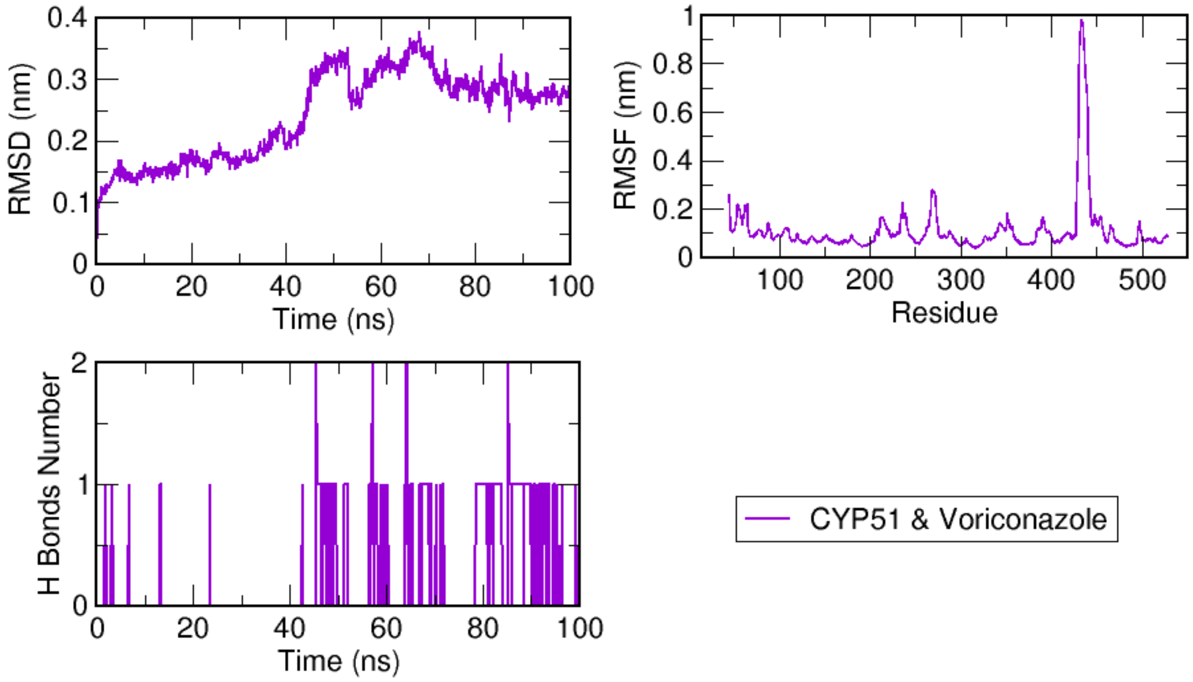

Figure S2. Root mean square deviation (RMSD), root mean square fluctuation (RMSF), and hydrogen bonds analysis of CYP51 & voriconazole for100 ns.

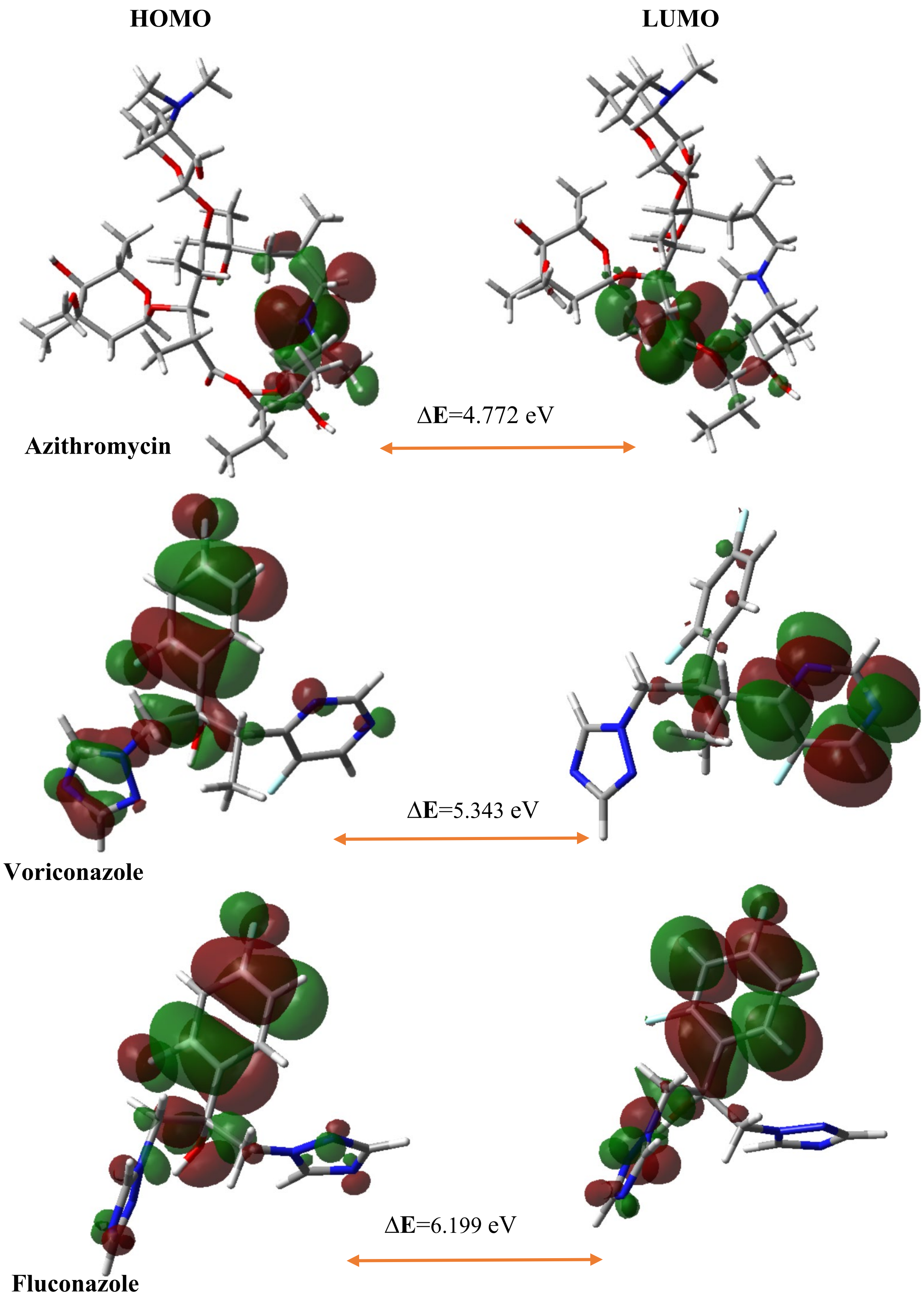

Figure S3. The HOMO (highest occupied molecular orbital) and the LUMO (lowest unoccupied molecular orbital) diagrams of standard compounds (**azithromycin**, **voriconazole** and **fluconazole**) calculated at B3LYP/6-311G(d,p) level.

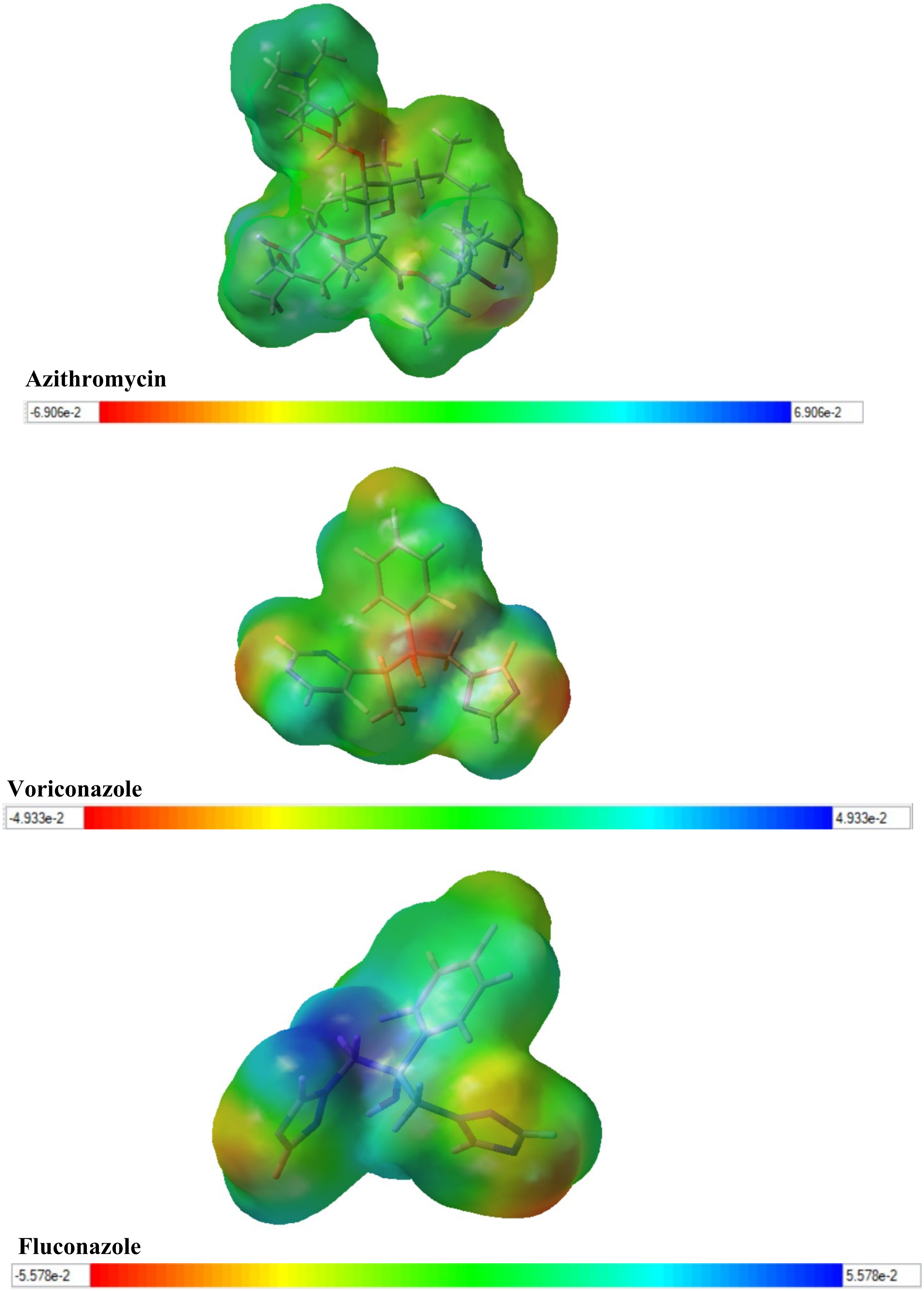

Figure S4. The Molecular electrostatic potential (MEP) diagrams of standard compounds (**azithromycin**, **voriconazole** and **fluconazole**) at B3LYP/6-311G(d,p) level.

5c

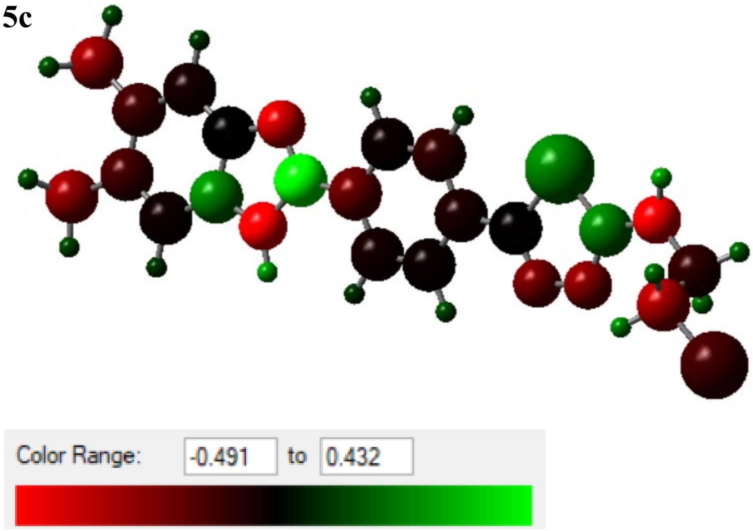

5f

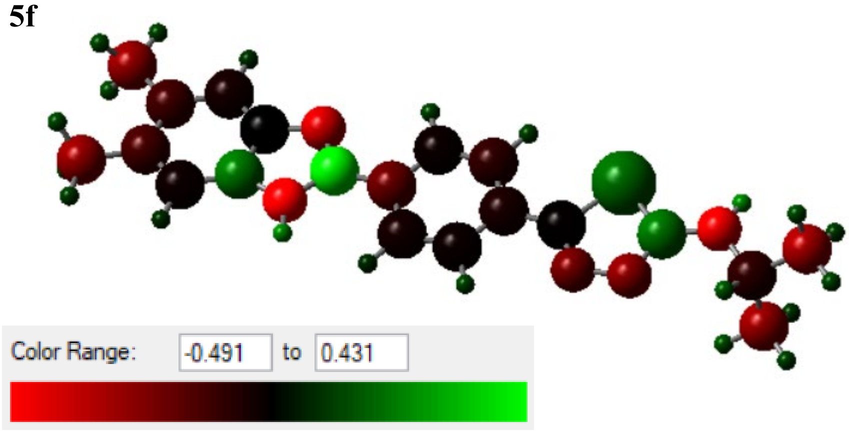

5h

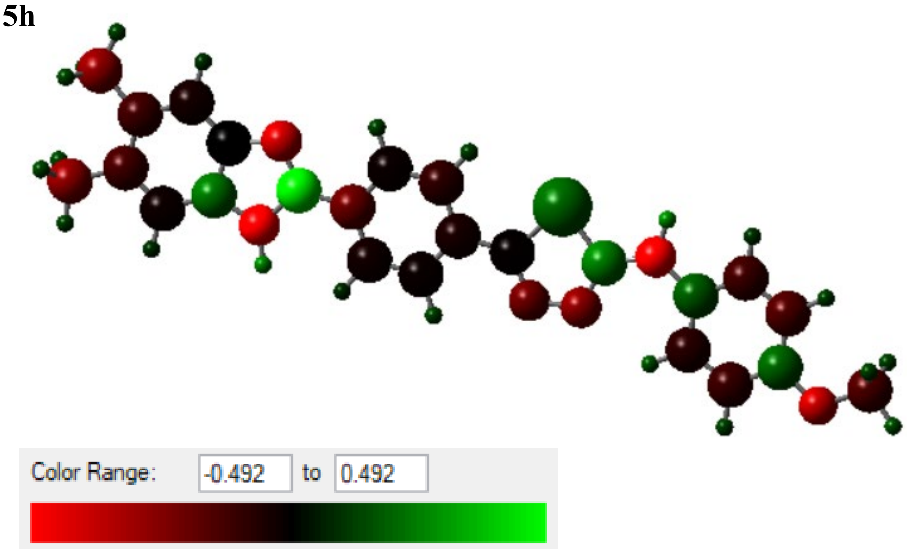

Figure S5. The mulliken atomic charge distrubition of **5c**, **5f** and **5h** molecules. Color ranges, in a.u.

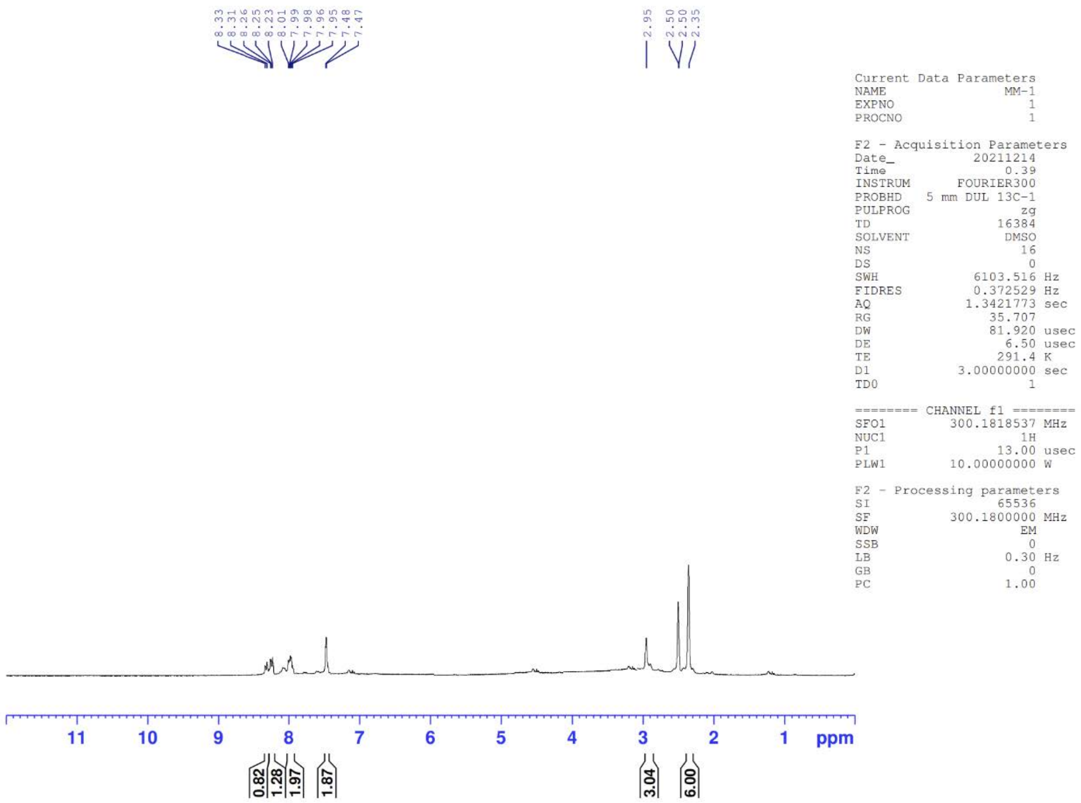

Figure S6. ^{1}H-NMR spectrum of compound **5a**

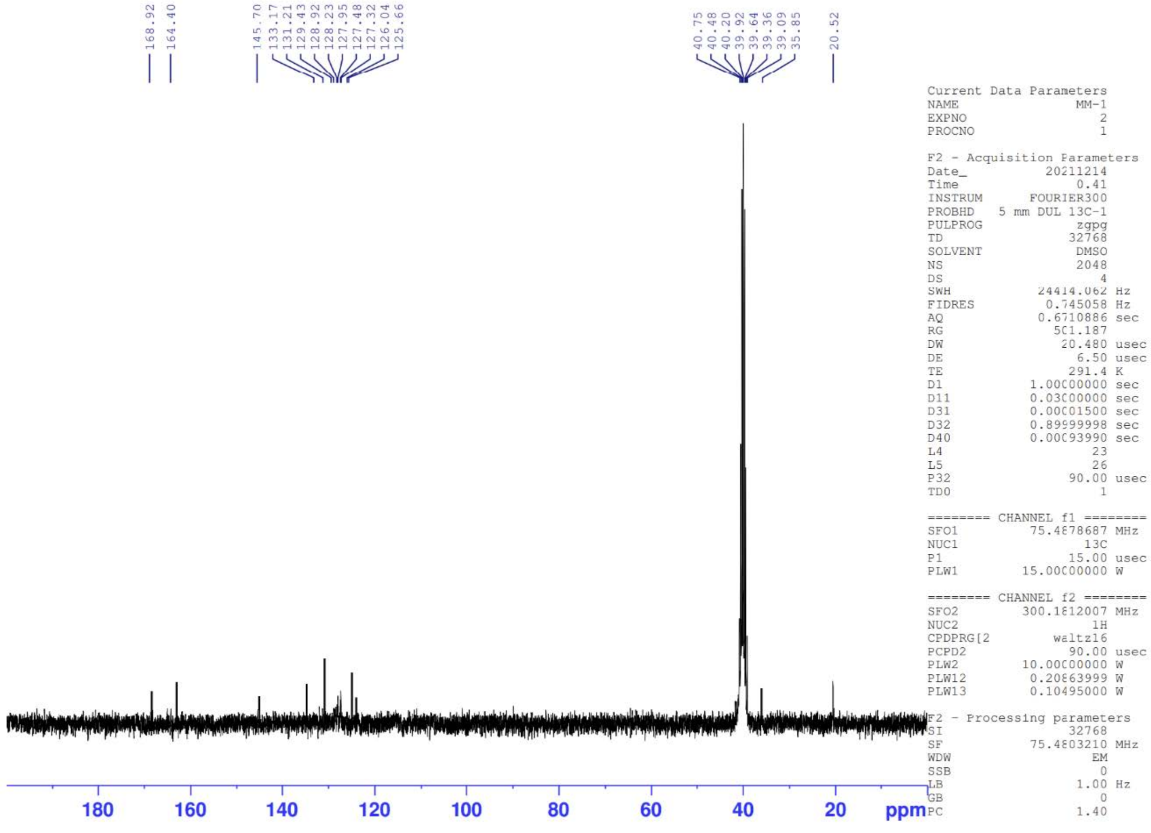

Figure S7. ^{13}C-NMR spectrum of compound **5a**

Data File: C:\LabSolutions\Data\Analiz\uac\MM-1_76.lcd

Elmt	Val.	Min	Max	Elmt	Val.	Min	Max	Elmt	Val.	Min	Max	Elmt	Val.	Min	Max	Use Adduct
H	1	10	40	O	2	0	4	S	2	1	1	Ru	2	0	0	H
C	4	9	40	F	1	0	0	Cl	1	0	0	Pd	2	0	0	
N	3	2	6	P	3	0	0	Br	1	0	0	I	3	0	0	

Error Margin (ppm):	5	DBE Range:	5.0 - 25.0	Electron Ions:	both
HC Ratio:	unlimited	Apply N Rule:	yes	Use MSn Info:	yes
Max Isotopes:	3	Isotope RI (%):	1.00	Isotope Res:	9000
MSn Iso RI (%):	10.00	MSn Logic Mode:	AND	Max Results:	150

Event#: 1 MS(E+) Ret. Time : 3.120 Scan# : 469

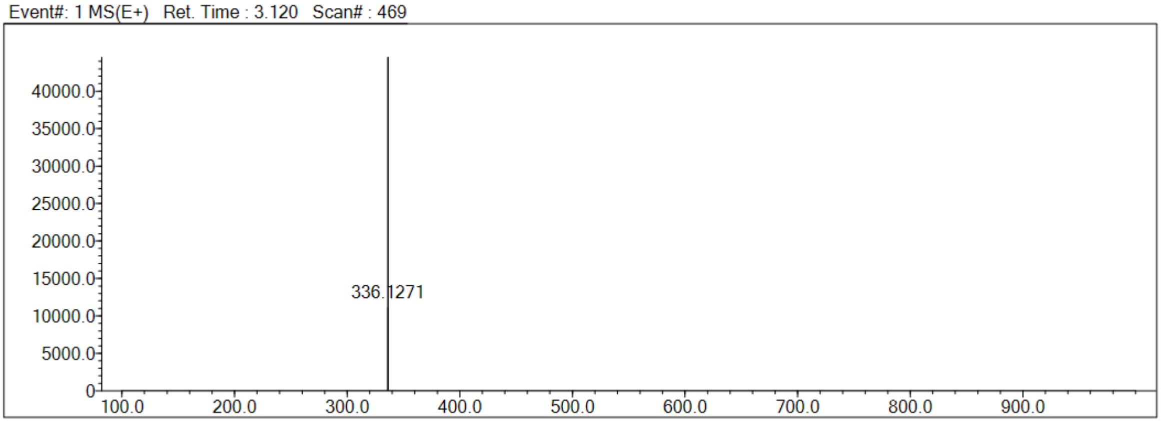

Measured region for 336.1271 m/z

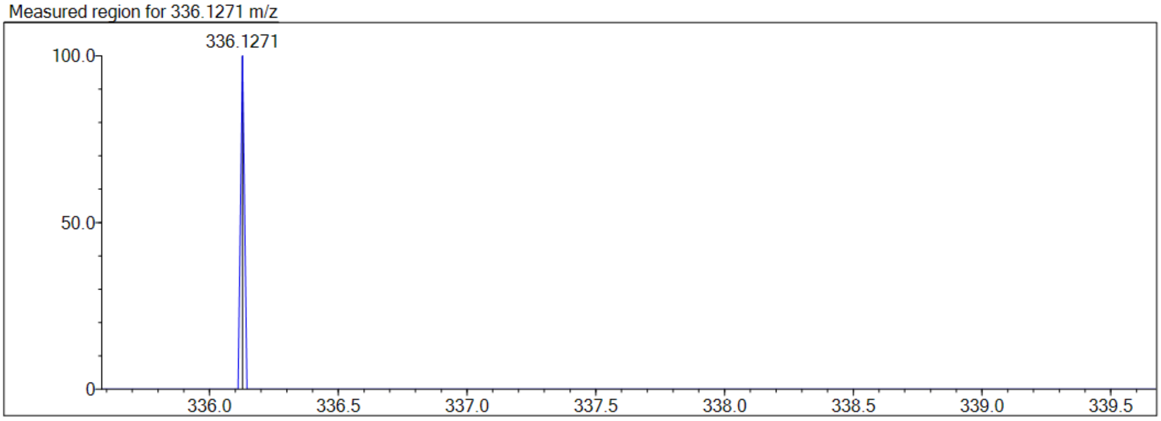

C18 H17 N5 S [M+H]+ : Predicted region for 336.1277 m/z

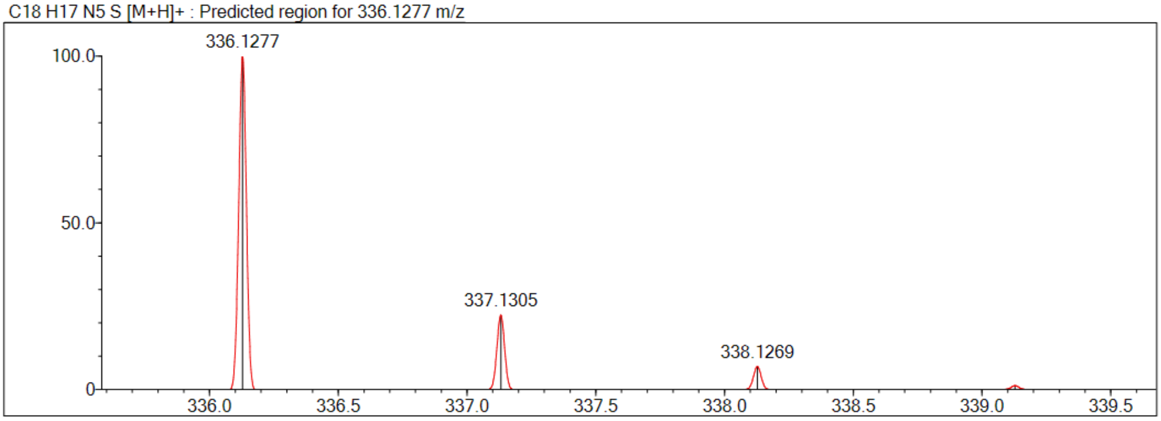

Rank	Score	Formula (M)	Ion	Meas. m/z	Pred. m/z	Df. (mDa)	Df. (ppm)	Iso	DBE
1	0.00	C18 H17 N5 S	[M+H]+	336.1271	336.1277	-0.6	-1.79	0.00	13.0

Figure S8. Mass spectrum of compound **5a**

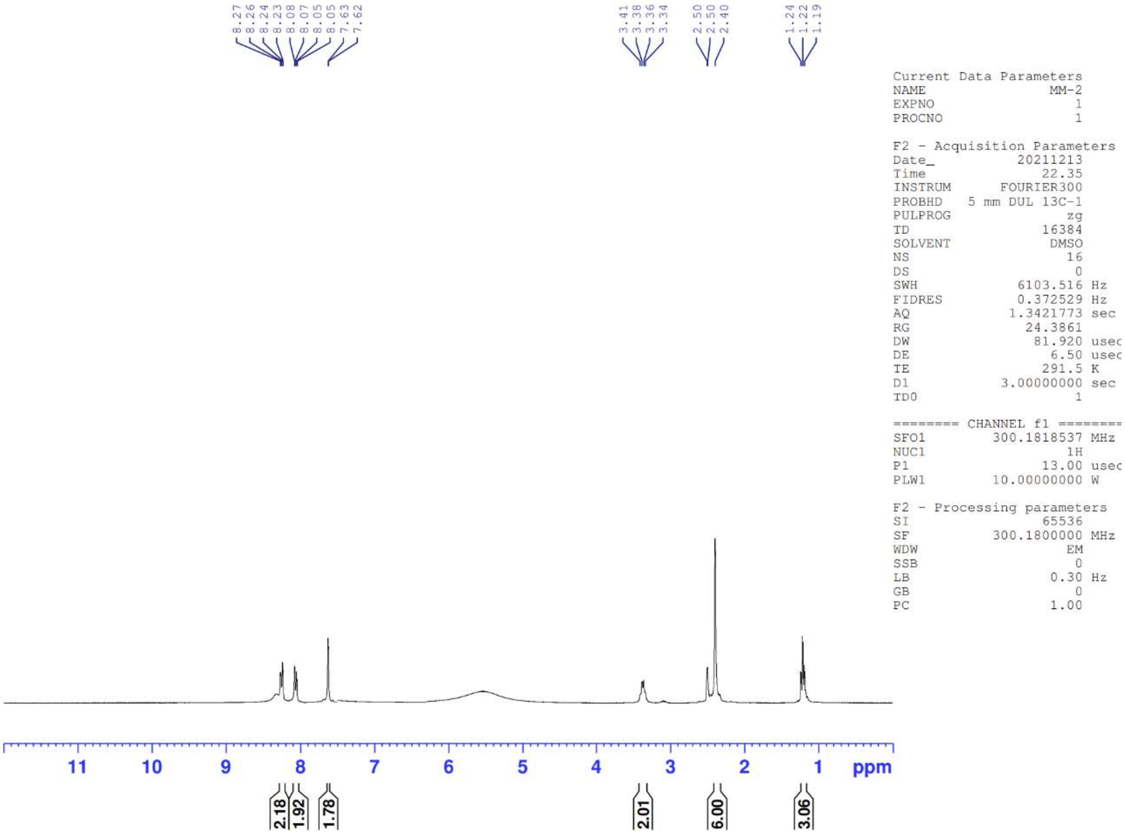

Figure S9. ^{1}H-NMR spectrum of compound **5b**

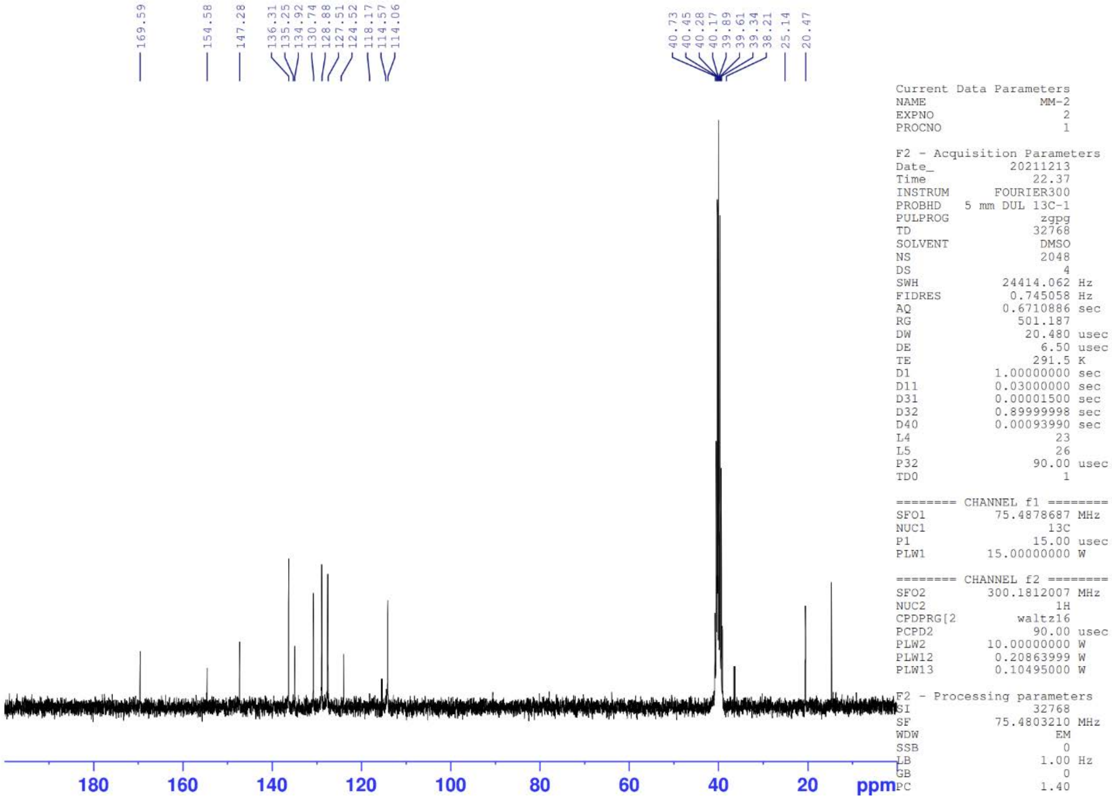

Figure S10. ^{13}C-NMR spectrum of compound **5b**

Data File: C:\LabSolutions\Data\Analiz\uac\MM-2_77.lcd

Elmt	Val.	Min	Max	Elmt	Val.	Min	Max	Elmt	Val.	Min	Max	Elmt	Val.	Min	Max	Use Adduct
H	1	10	40	O	2	0	4	S	2	1	1	Ru	2	0	0	H
C	4	9	40	F	1	0	0	Cl	1	0	0	Pd	2	0	0	
N	3	2	6	P	3	0	0	Br	1	0	0	I	3	0	0	

Error Margin (ppm): 5
HC Ratio: unlimited
Max Isotopes: 3
MSn Iso RI (%): 10.00

DBE Range: 5.0 - 20.0
Apply N Rule: yes
Isotope RI (%): 1.00
MSn Logic Mode: AND

Electron Ions: both
Use MSn Info: yes
Isotope Res: 9000
Max Results: 150

Event#: 1 MS(E+) Ret. Time : 3.360 Scan# : 505

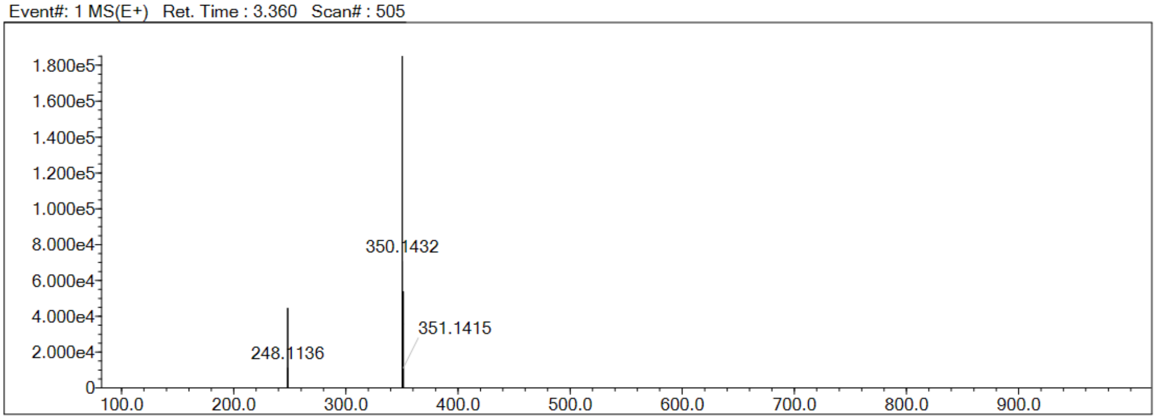

Measured region for 350.1432 m/z

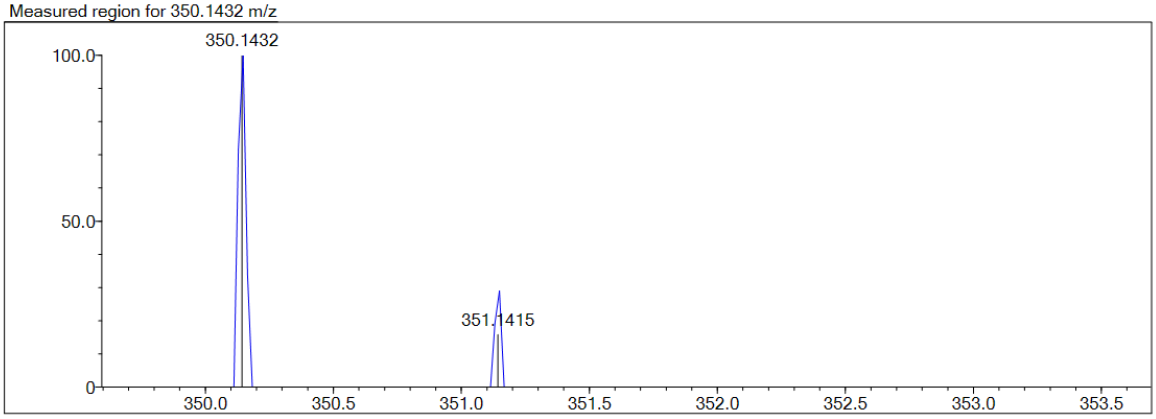

C19 H19 N5 S [M+H]+ : Predicted region for 350.1434 m/z

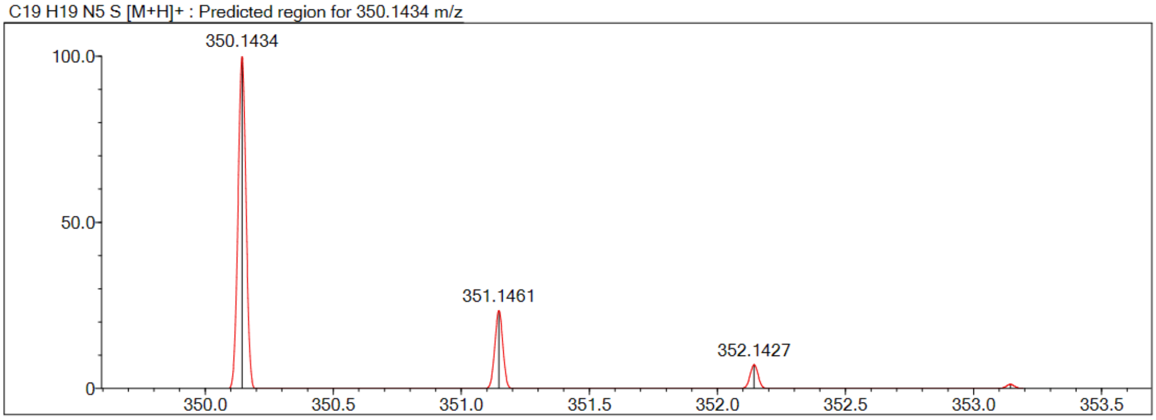

Rank	Score	Formula (M)	Ion	Meas. m/z	Pred. m/z	Df. (mDa)	Df. (ppm)	Iso	DBE
1	0.00	C19 H19 N5 S	[M+H]+	350.1432	350.1434	-0.2	-0.57	0.00	13.0

Figure S11. Mass spectrum of compound **5b**

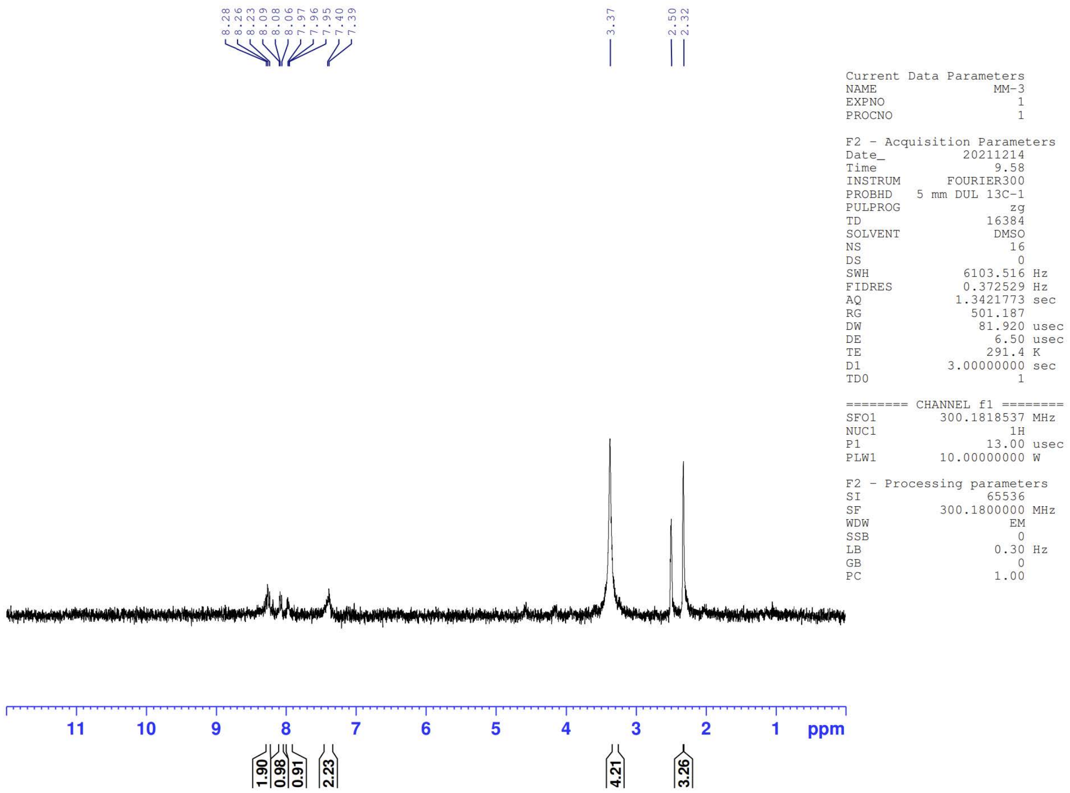

Figure S12. ^{1}H-NMR spectrum of compound **5c**

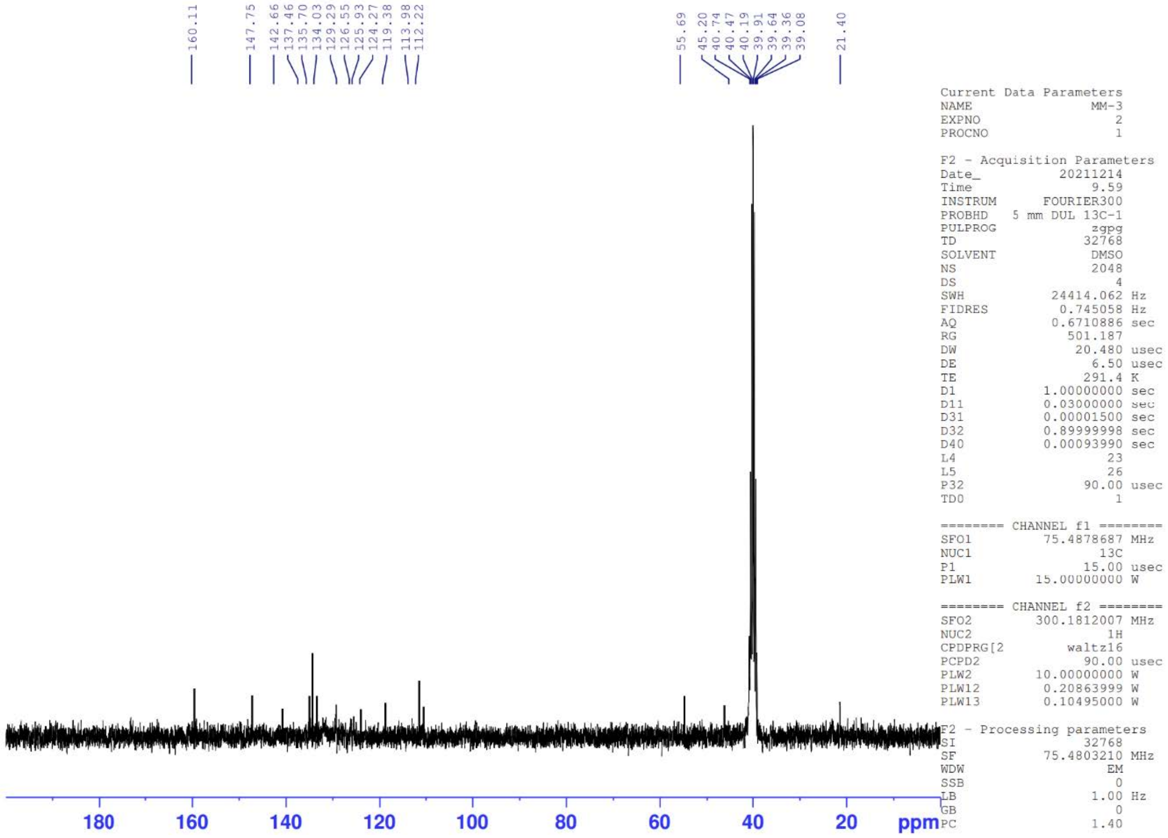

Figure S13. ^{13}C-NMR spectrum of compound **5c**

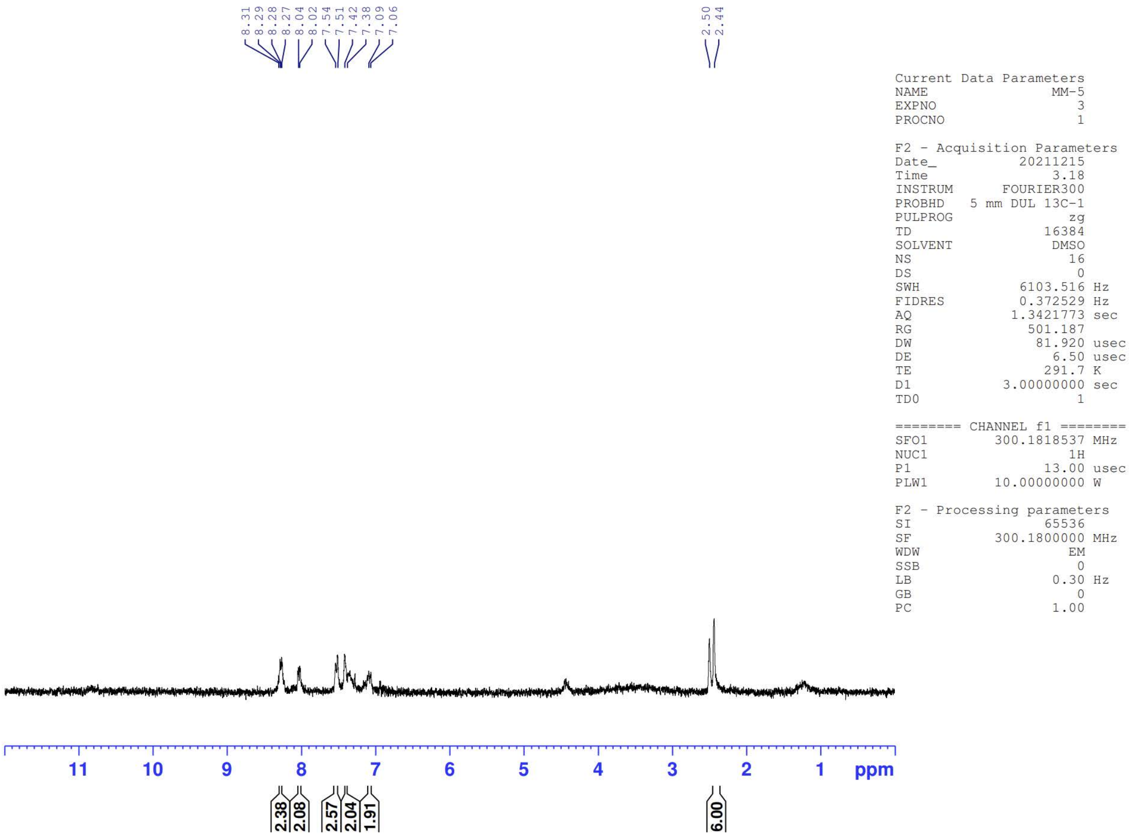

Figure S14. ^{1}H-NMR spectrum of compound **5d**

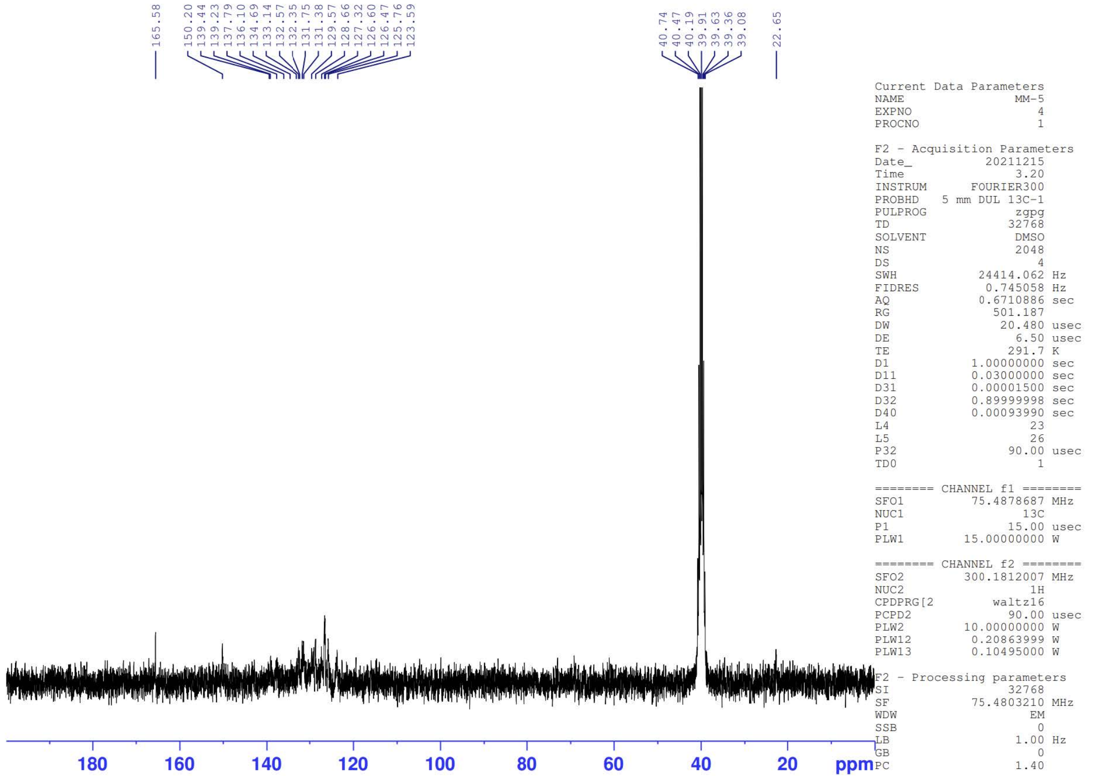

Figure S15. ^{13}C-NMR spectrum of compound **5d**

Data File: C:\LabSolutions\Data\Analiz\uac\MM-5_185.lcd

Elmt	Val.	Min	Max	Elmt	Val.	Min	Max	Elmt	Val.	Min	Max	Elmt	Val.	Min	Max	Use Adduct
H	1	0	25	O	2	0	4	Cl	1	1	1	I	3	0	0	H
B	3	0	0	F	1	0	0	Br	1	0	0					
C	4	0	35	P	3	0	0	Ru	2	0	0					
N	3	0	5	S	2	0	1	Pd	2	0	0					

Error Margin (ppm): 5
HC Ratio: unlimited
Max Isotopes: 3
MSn Iso RI (%): 10.00

DBE Range: 5.0 - 25.0
Apply N Rule: yes
Isotope RI (%): 1.00
MSn Logic Mode: AND

Electron Ions: both
Use MSn Info: yes
Isotope Res: 9000
Max Results: 50

Event#: 1 MS(E+) Ret. Time : 2.747 Scan# : 413

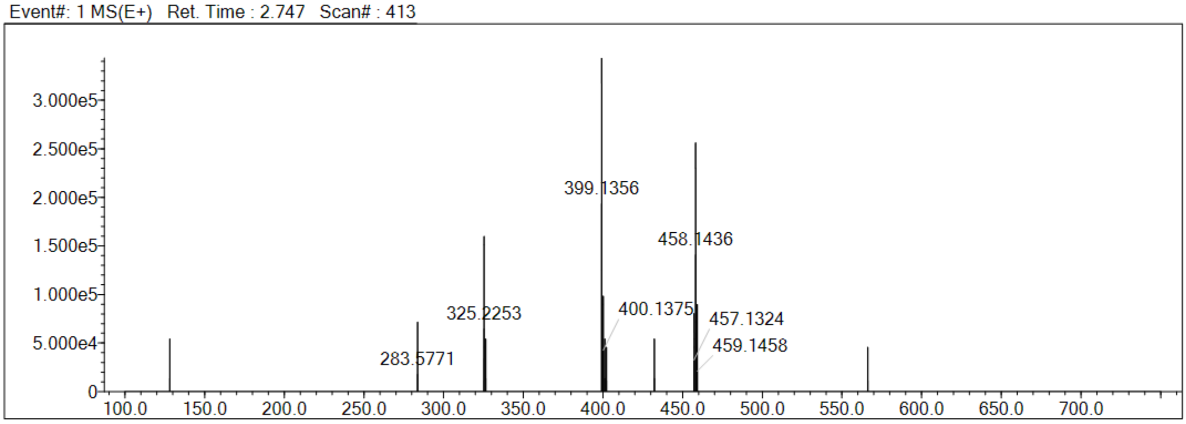

Measured region for 432.1059 m/z

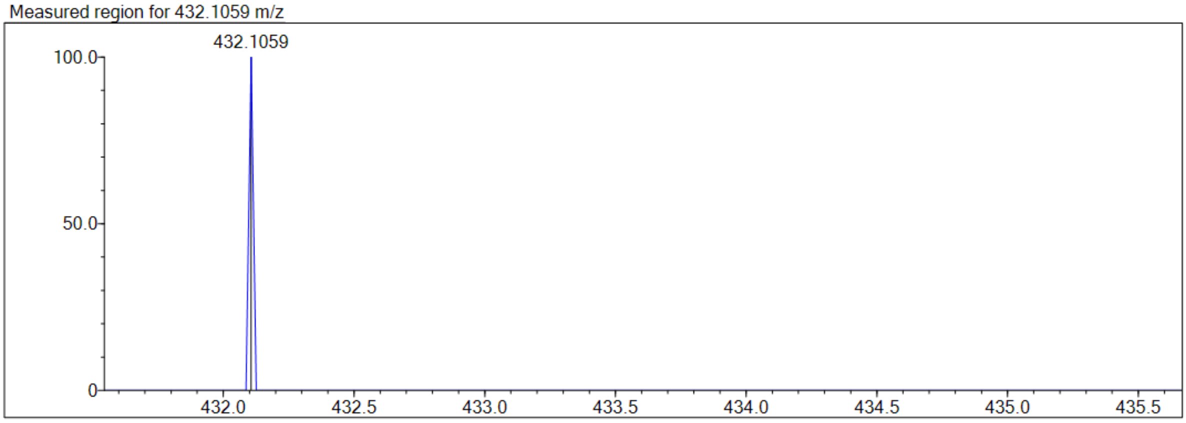

C23 H18 N5 S Cl [M+H]+ : Predicted region for 432.1044 m/z

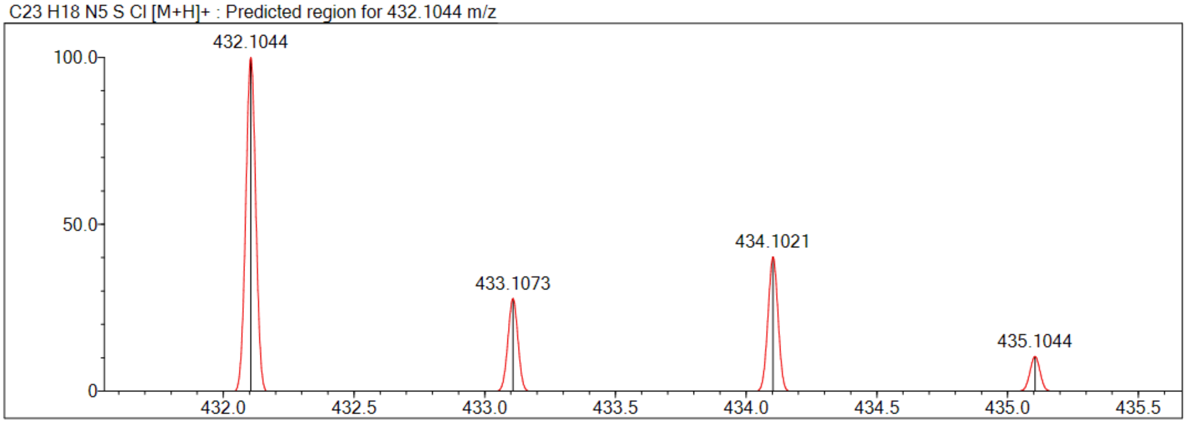

Rank	Score	Formula (M)	Ion	Meas. m/z	Pred. m/z	Df. (mDa)	Df. (ppm)	Iso	DBE
1	0.00	C23 H18 N5 S Cl	[M+H]+	432.1059	432.1044	1.5	3.47	0.00	17.0

Figure S16. Mass spectrum of compound **5d**

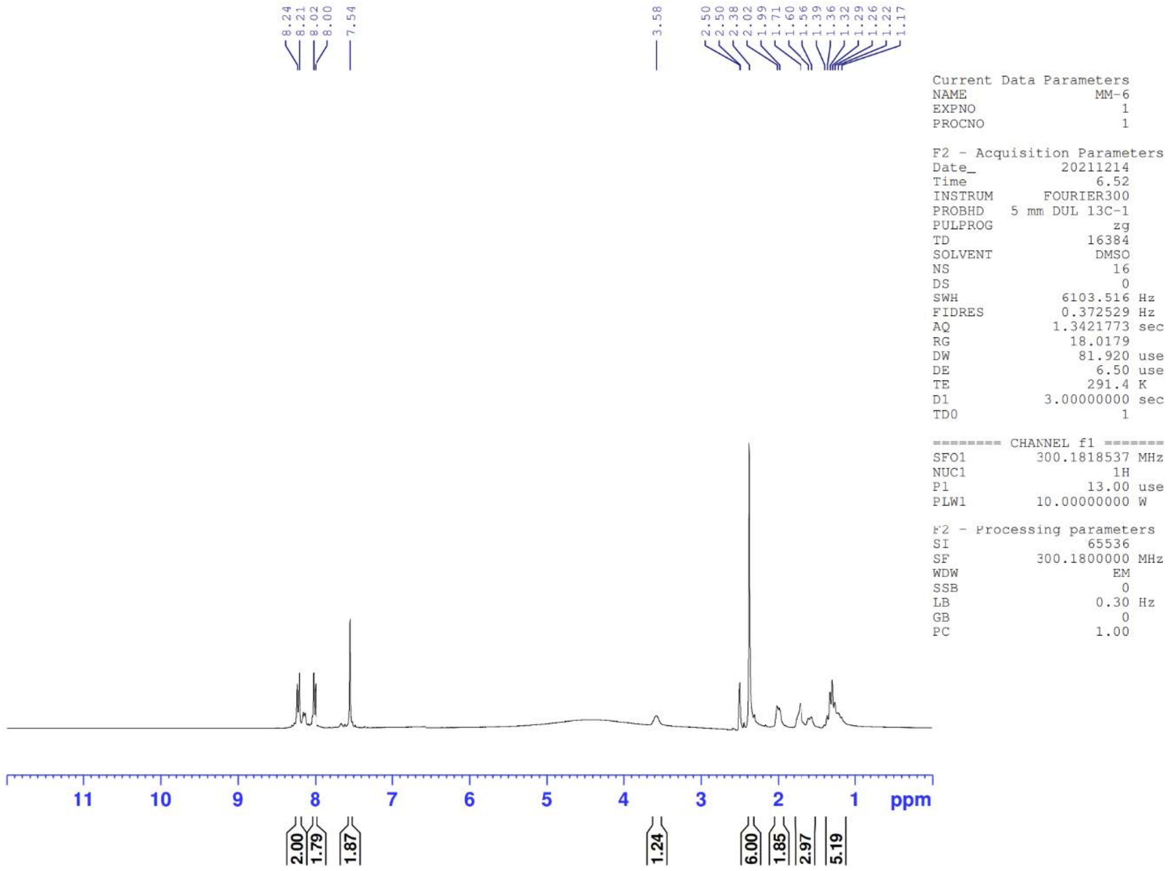

Figure S17. ^{1}H-NMR spectrum of compound **5e**

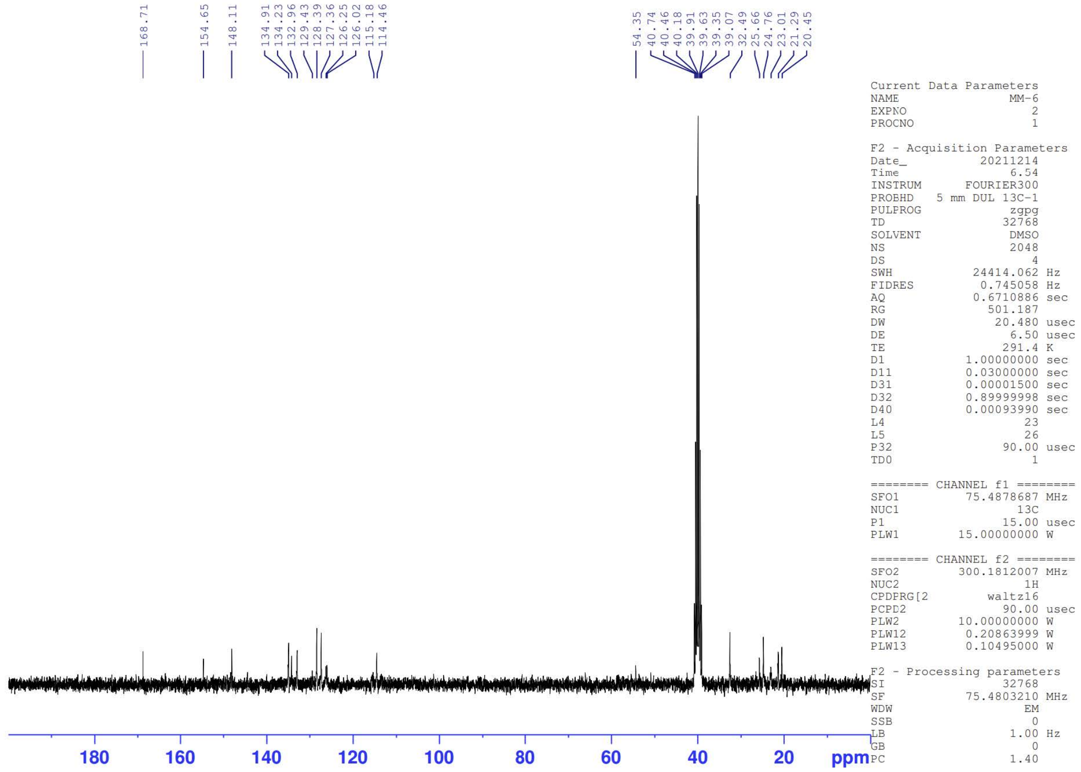

Figure S18. ^{13}C-NMR spectrum of compound **5e**

Data File: C:\LabSolutions\Data\Analiz\uac\MM-6_186.lcd

Elmt	Val.	Min	Max	Elmt	Val.	Min	Max	Elmt	Val.	Min	Max	Elmt	Val.	Min	Max	Use Adduct
H	1	0	25	O	2	0	4	Cl	1	0	0	I	3	0	0	H
B	3	0	0	F	1	0	0	Br	1	0	0					
C	4	0	35	P	3	0	0	Ru	2	0	0					
N	3	0	5	S	2	0	1	Pd	2	0	0					

Error Margin (ppm): 5
HC Ratio: unlimited
Max Isotopes: 3
MSn Iso RI (%): 10.00

DBE Range: 5.0 - 25.0
Apply N Rule: yes
Isotope RI (%): 1.00
MSn Logic Mode: AND

Electron Ions: both
Use MSn Info: yes
Isotope Res: 9000
Max Results: 50

Event#: 1 MS(E+) Ret. Time : 2.867 Scan# : 431

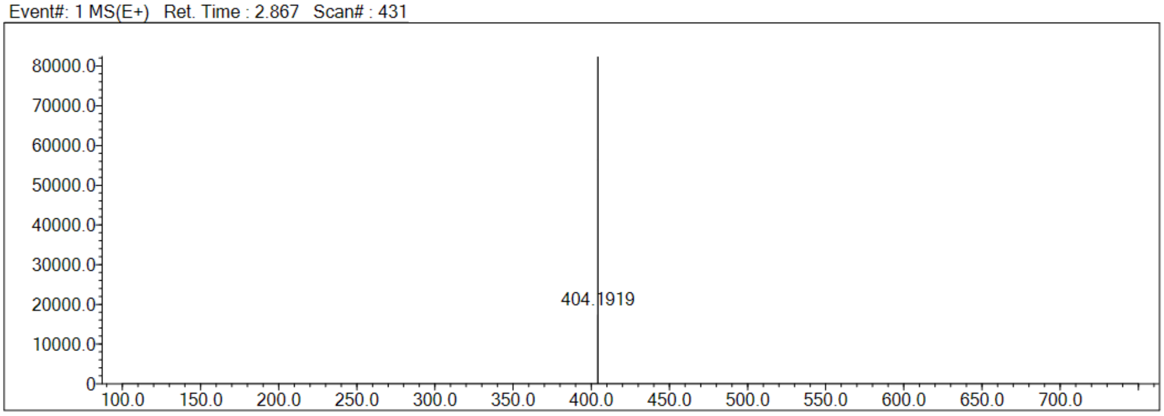

Measured region for 404.1919 m/z

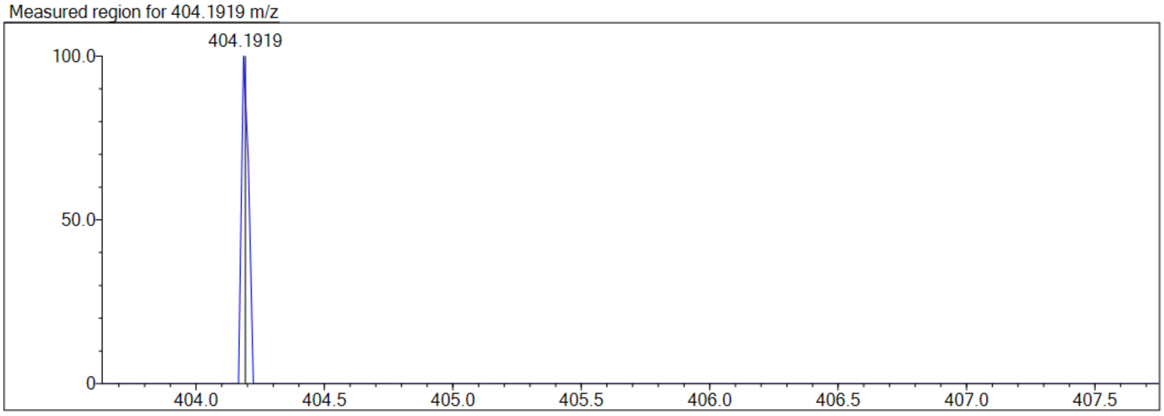

C23 H25 N5 S [M+H]+ : Predicted region for 404.1903 m/z

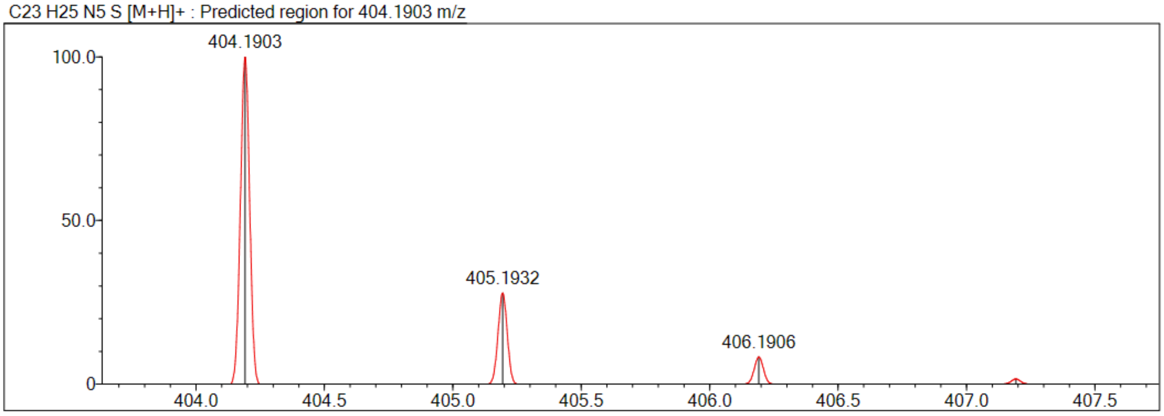

Rank	Score	Formula (M)	Ion	Meas. m/z	Pred. m/z	Df. (mDa)	Df. (ppm)	Iso	DBE
1	0.00	C23 H25 N5 S	[M+H]+	404.1919	404.1903	1.6	3.96	0.00	14.0

Figure S19. Mass spectrum of compound **5e**

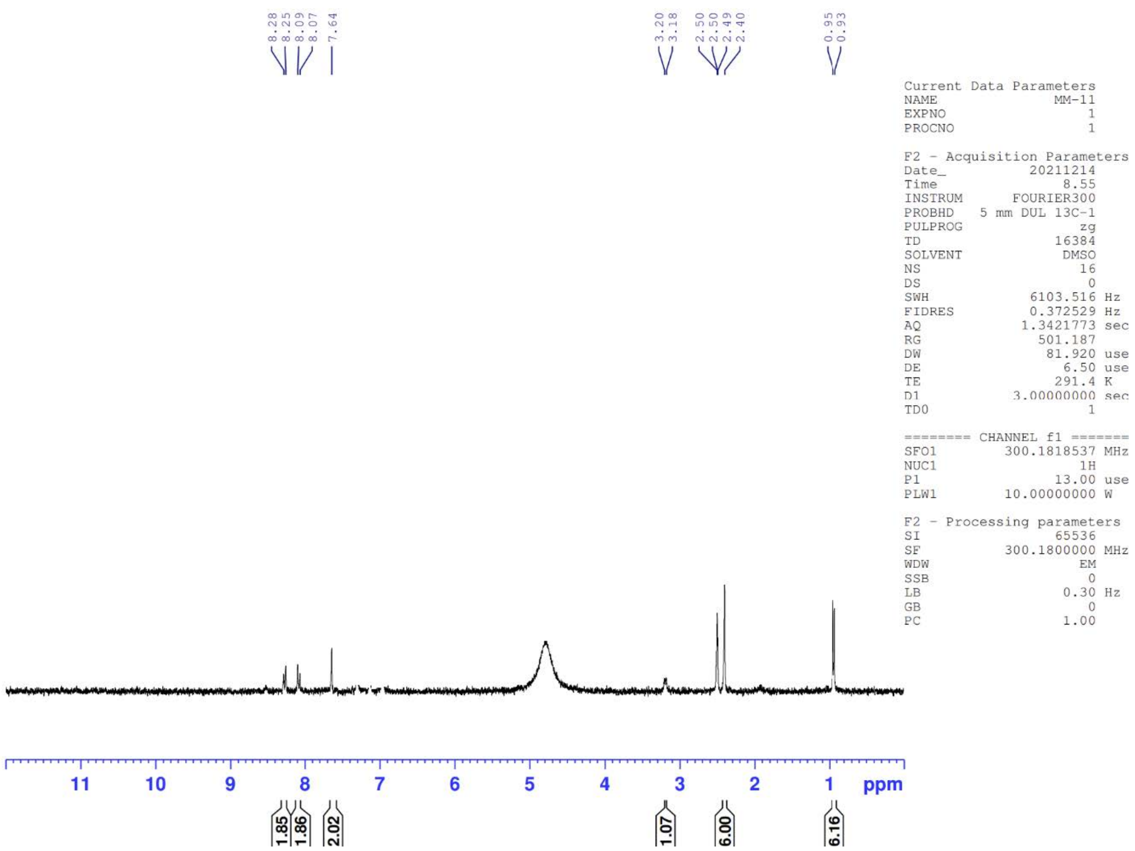

Figure S20. ^{1}H-NMR spectrum of compound **5f**

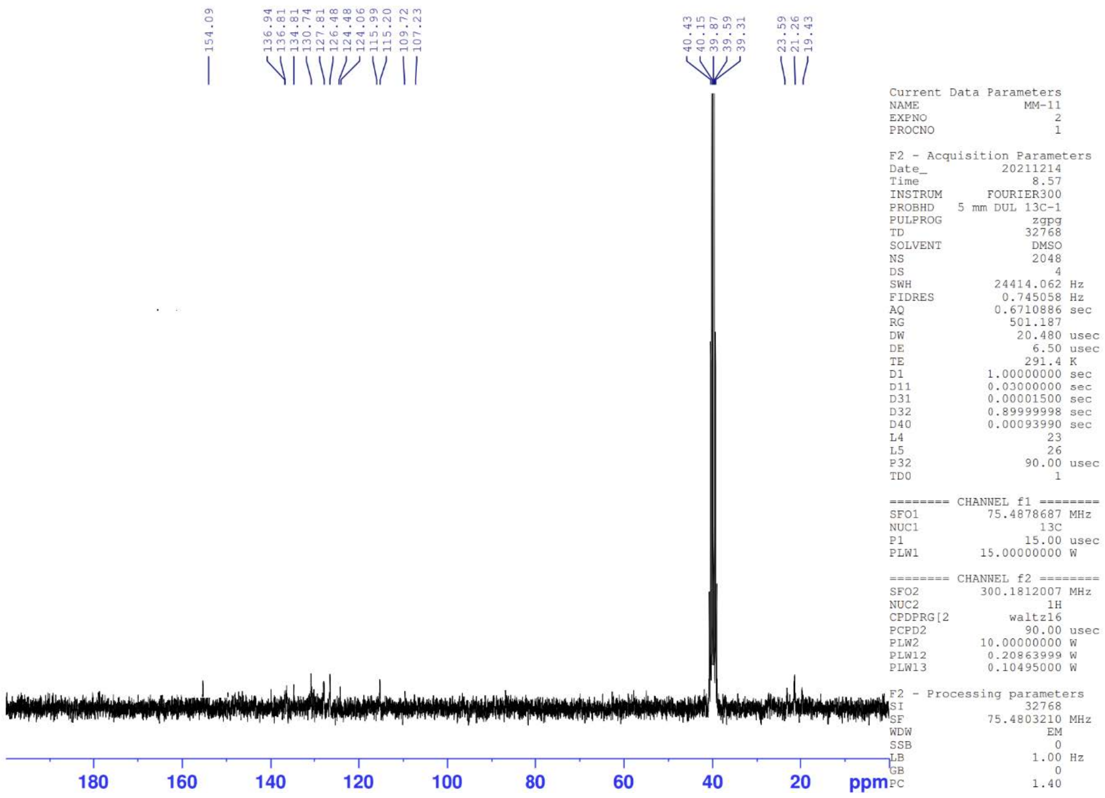

Figure S21. ^{13}C-NMR spectrum of compound **5f**

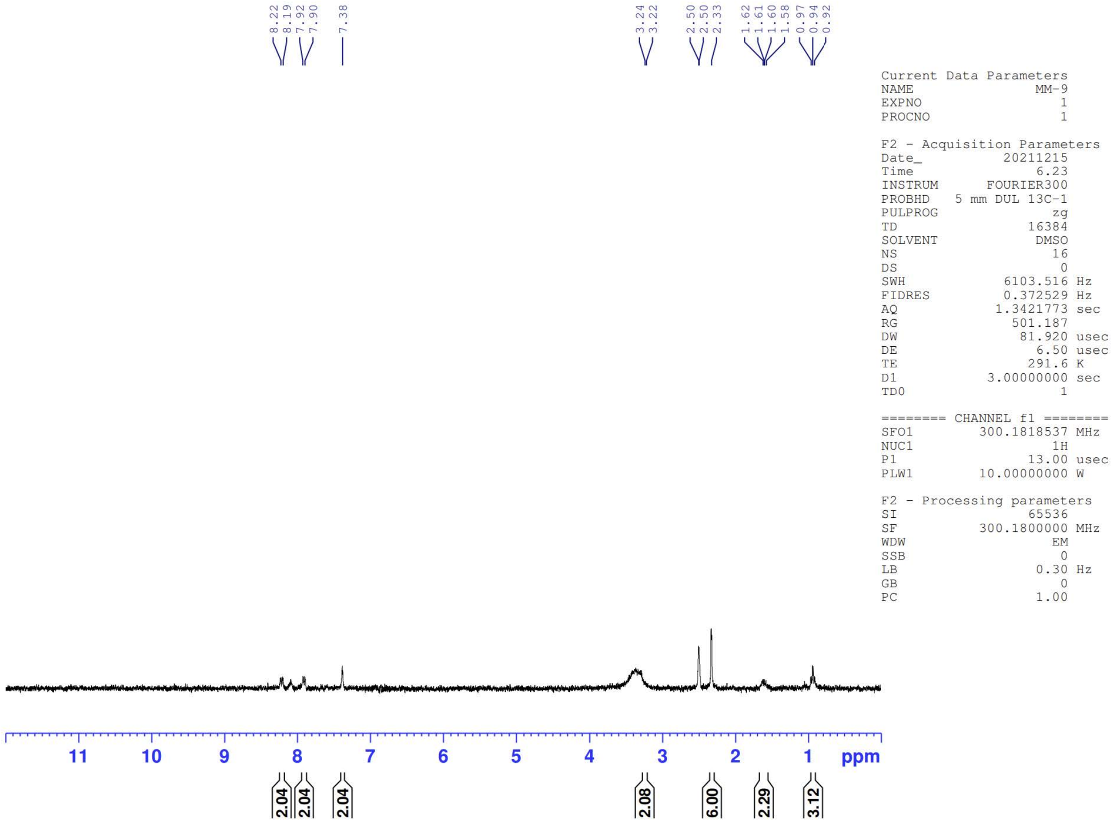

Figure S22. ^{1}H-NMR spectrum of compound **5g**

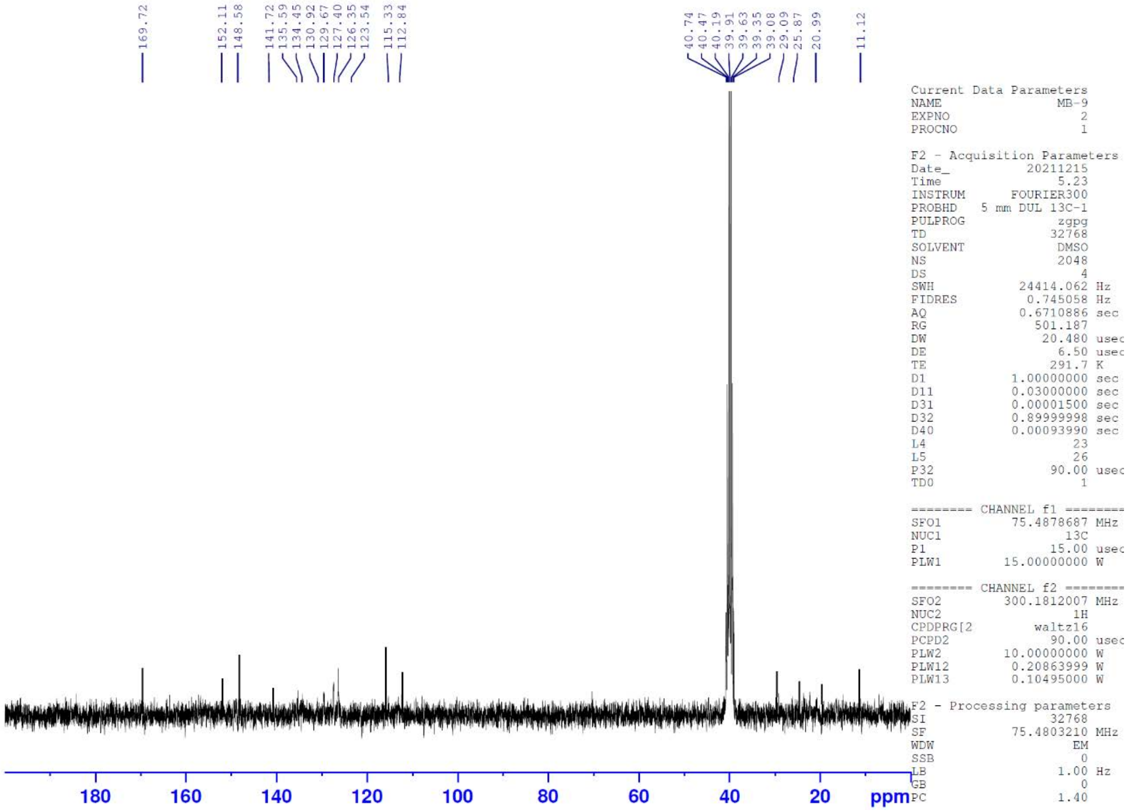

Figure S23. ^{13}C-NMR spectrum of compound **5g**

Formula Predictor Report - MM-9_83.lcd　　Page 1 of 1

Data File: C:\LabSolutions\Data\Analiz\uac\MM-9_83.lcd

Elmt	Val.	Min	Max	Elmt	Val.	Min	Max	Elmt	Val.	Min	Max	Elmt	Val.	Min	Max	Use Adduct
H	1	10	40	O	2	0	4	S	2	1	1	Ru	2	0	0	H
C	4	9	40	F	1	0	0	Cl	1	0	0	Pd	2	0	0	
N	3	2	6	P	3	0	0	Br	1	0	0	I	3	0	0	

Error Margin (ppm):	5	DBE Range:	5.0 - 25.0	Electron Ions:	both
HC Ratio:	unlimited	Apply N Rule:	yes	Use MSn Info:	yes
Max Isotopes:	3	Isotope RI (%):	1.00	Isotope Res:	9000
MSn Iso RI (%):	10.00	MSn Logic Mode:	AND	Max Results:	150

Event#: 1 MS(E+)　Ret. Time : 3.347　Scan# : 503

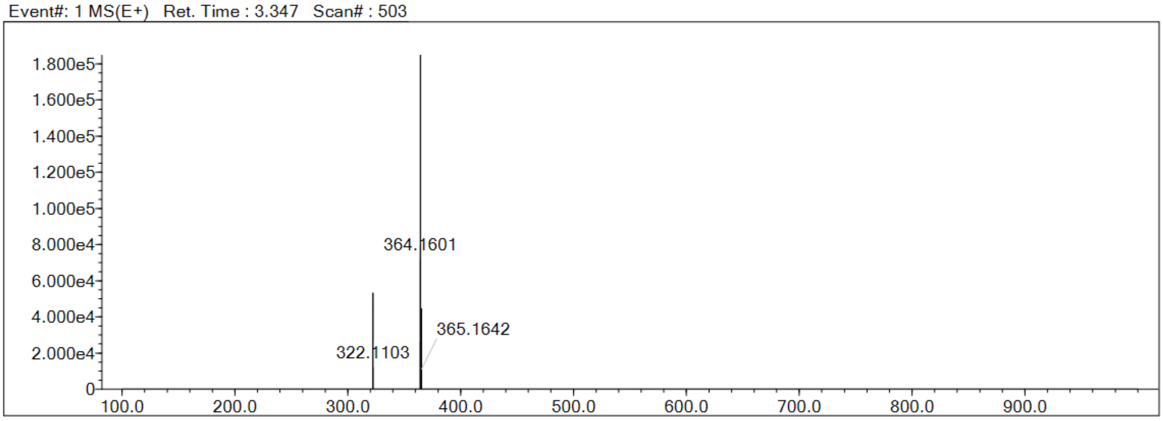

Measured region for 364.1601 m/z

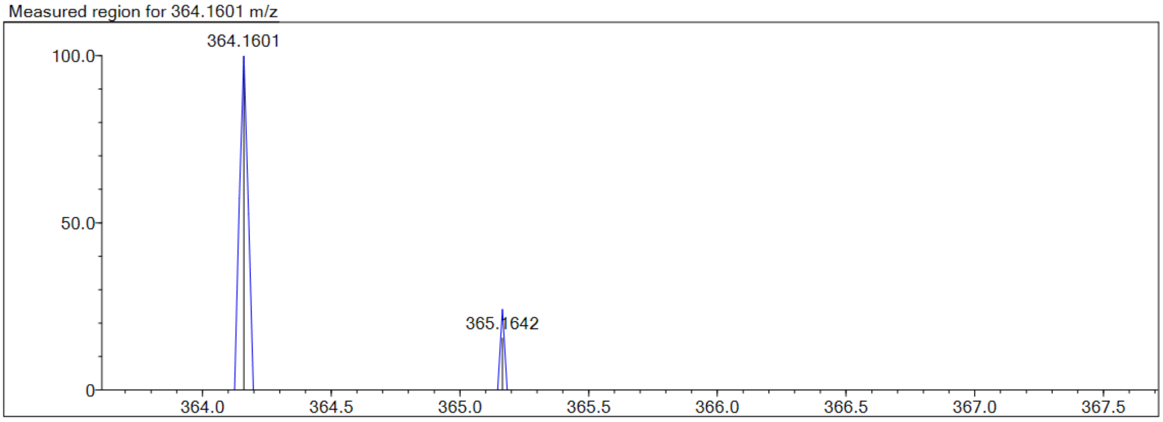

C20 H21 N5 S [M+H]+ : Predicted region for 364.1590 m/z

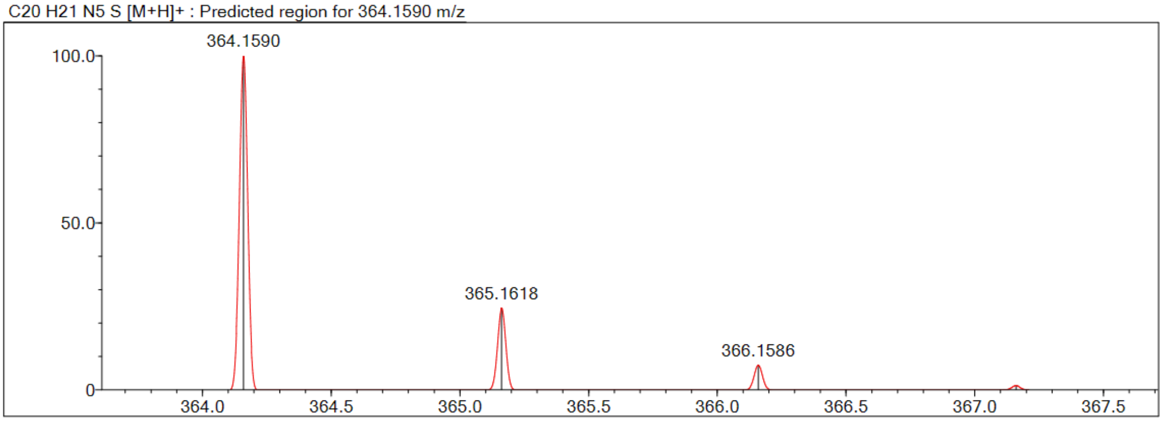

Rank	Score	Formula (M)	Ion	Meas. m/z	Pred. m/z	Df. (mDa)	Df. (ppm)	Iso	DBE
1	0.00	C20 H21 N5 S	[M+H]+	364.1601	364.1590	1.1	3.02	0.00	13.0

Figure S24. Mass spectrum of compound **5g**

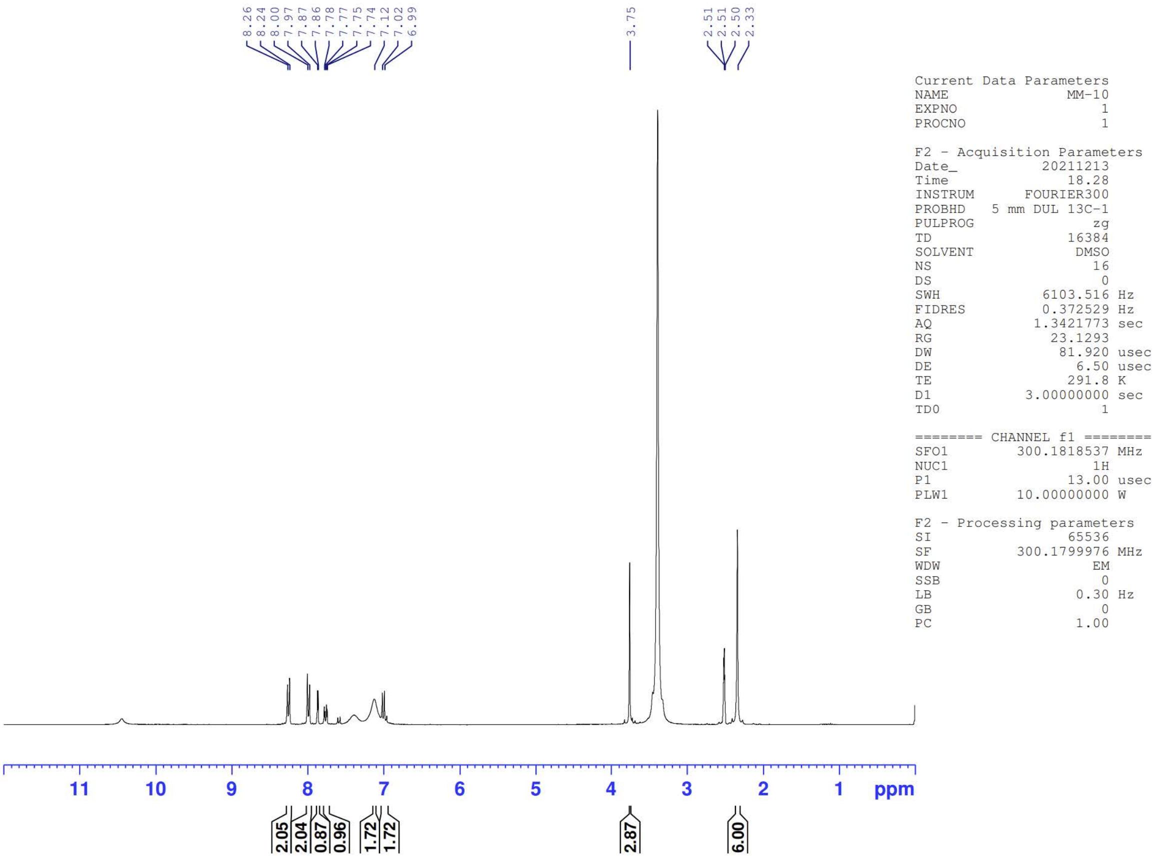

Figure S25. ^{1}H-NMR spectrum of compound **5h**

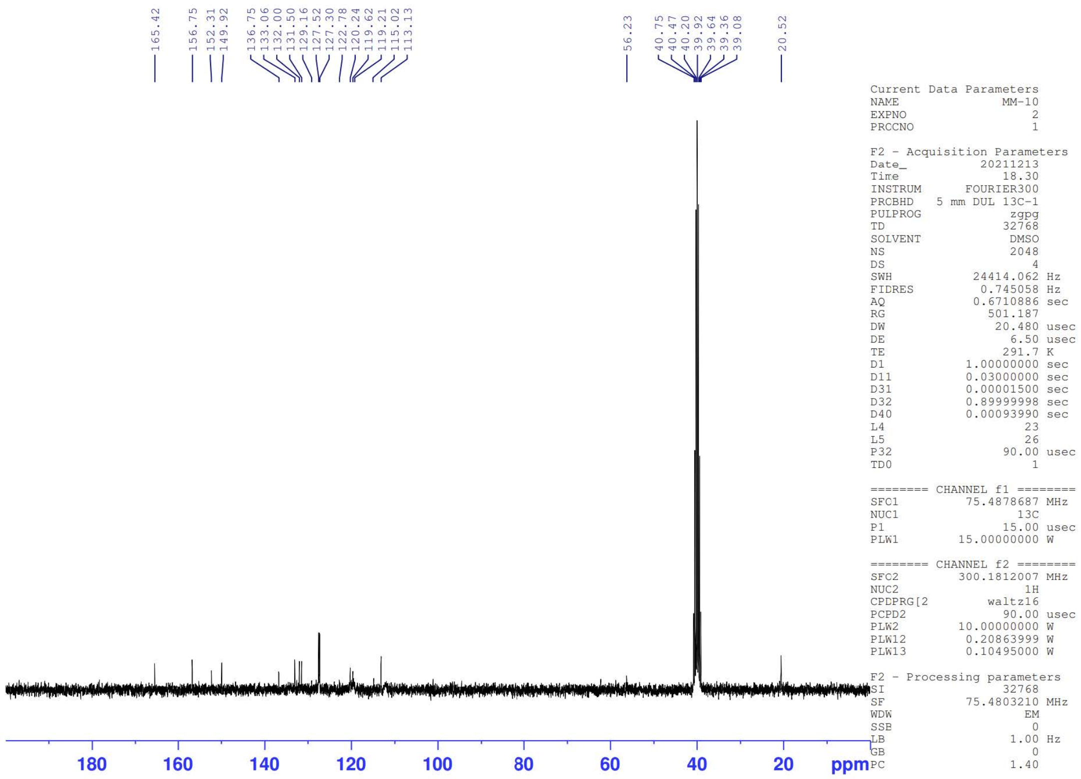

Figure S26. ^{13}C-NMR spectrum of compound **5h**

Data File: C:\LabSolutions\Data\Analiz\uac\MM-10_188.lcd

Elmt	Val.	Min	Max	Elmt	Val.	Min	Max	Elmt	Val.	Min	Max	Elmt	Val.	Min	Max	Use Adduct
H	1	0	25	O	2	0	4	Cl	1	0	0	I	3	0	0	H
B	3	0	0	F	1	0	0	Br	1	0	0					
C	4	0	35	P	3	0	0	Ru	2	0	0					
N	3	0	5	S	2	0	1	Pd	2	0	0					

Error Margin (ppm): 5
HC Ratio: unlimited
Max Isotopes: 3
MSn Iso RI (%): 10.00

DBE Range: 5.0 - 25.0
Apply N Rule: yes
Isotope RI (%): 1.00
MSn Logic Mode: AND

Electron Ions: both
Use MSn Info: yes
Isotope Res: 9000
Max Results: 50

Event#: 1 MS(E+) Ret. Time : 2.413 Scan# : 363

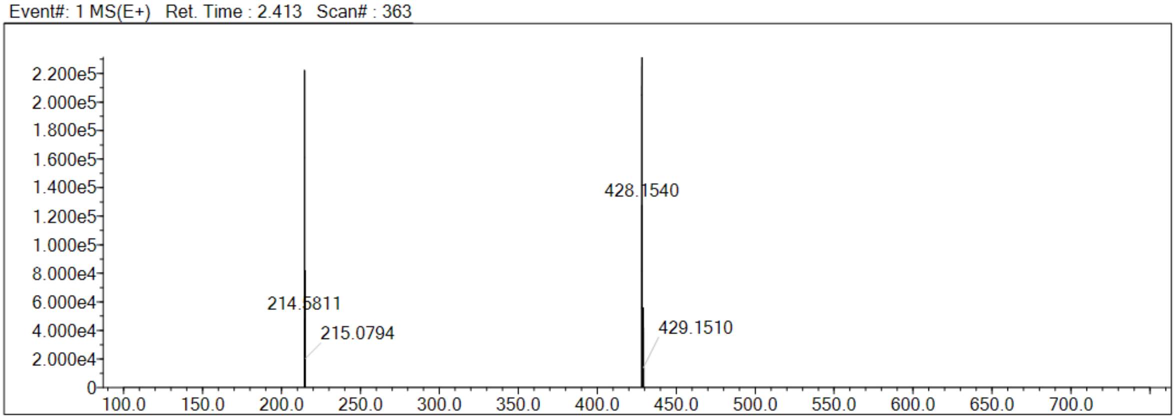

Measured region for 428.1540 m/z

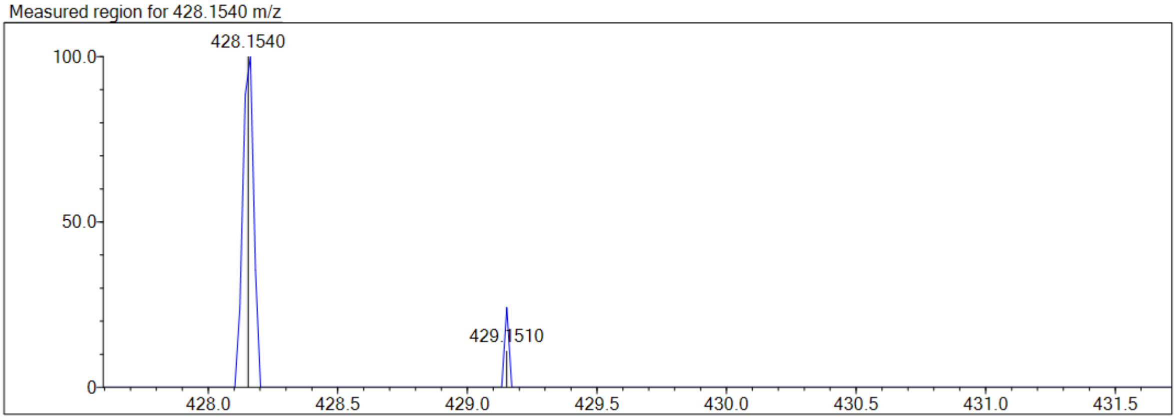

C24 H21 N5 O S [M+H]+ : Predicted region for 428.1540 m/z

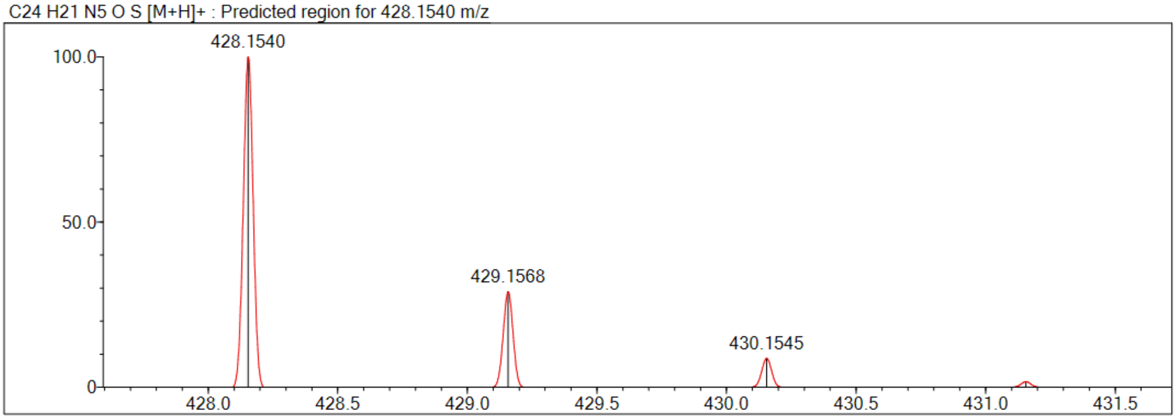

Rank	Score	Formula (M)	Ion	Meas. m/z	Pred. m/z	Df. (mDa)	Df. (ppm)	Iso	DBE
1	0.00	C24 H21 N5 O S	[M+H]+	428.1540	428.1540	0.0	0.00	0.00	17.0

Figure S27. Mass spectrum of compound **5h**
