# Supplementary material for: Synthesis, Molecular Docking, Dynamics, Quantum-Chemical Computation, and Antimicrobial Activity Studies of Some New Benzimidazole–Thiadiazole Hybrids
Source: ACS Omega. 2022 Dec 9;7(50):47015–30. doi: 10.1021/acsomega.2c06142 (PMC9773947; doi:10.1021/acsomega.2c06142)
Supplement: Supplementary file 2 — ao2c06142_si_002.zip [file ao2c06142_si_002.zip › Supporting Information-2.pptx]

## Slide 1
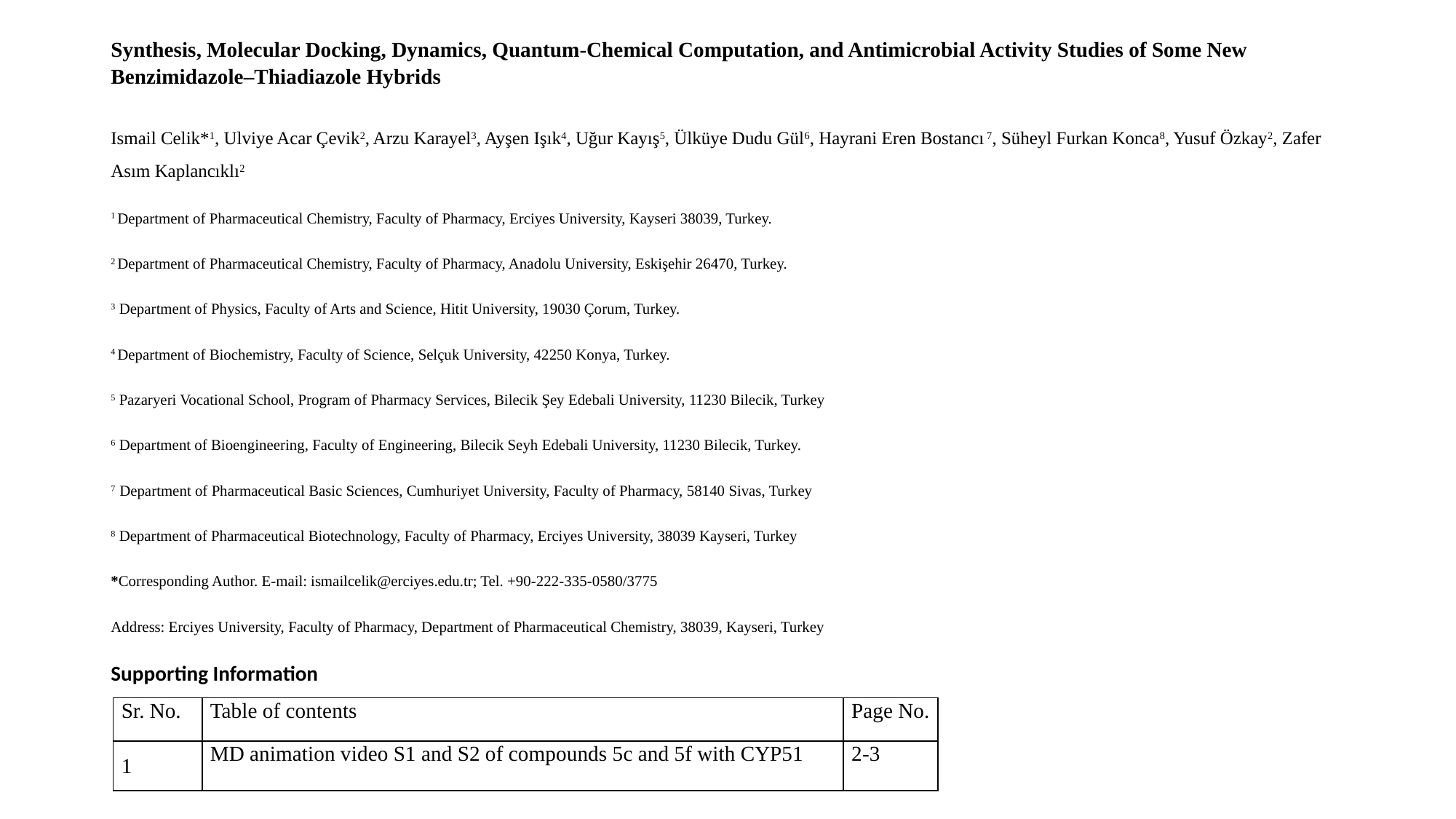

Synthesis, Molecular Docking, Dynamics, Quantum-Chemical Computation, and Antimicrobial Activity Studies of Some New Benzimidazole–Thiadiazole Hybrids
Ismail Celik*1, Ulviye Acar Çevik2, Arzu Karayel3, Ayşen Işık4, Uğur Kayış5, Ülküye Dudu Gül6, Hayrani Eren Bostancı 7, Süheyl Furkan Konca8, Yusuf Özkay2, Zafer Asım Kaplancıklı2
1 Department of Pharmaceutical Chemistry, Faculty of Pharmacy, Erciyes University, Kayseri 38039, Turkey.
2 Department of Pharmaceutical Chemistry, Faculty of Pharmacy, Anadolu University, Eskişehir 26470, Turkey.
3 Department of Physics, Faculty of Arts and Science, Hitit University, 19030 Çorum, Turkey.
4 Department of Biochemistry, Faculty of Science, Selçuk University, 42250 Konya, Turkey.
5 Pazaryeri Vocational School, Program of Pharmacy Services, Bilecik Şey Edebali University, 11230 Bilecik, Turkey
6 Department of Bioengineering, Faculty of Engineering, Bilecik Seyh Edebali University, 11230 Bilecik, Turkey.
7 Department of Pharmaceutical Basic Sciences, Cumhuriyet University, Faculty of Pharmacy, 58140 Sivas, Turkey
8 Department of Pharmaceutical Biotechnology, Faculty of Pharmacy, Erciyes University, 38039 Kayseri, Turkey
*Corresponding Author. E-mail: ismailcelik@erciyes.edu.tr; Tel. +90-222-335-0580/3775
Address: Erciyes University, Faculty of Pharmacy, Department of Pharmaceutical Chemistry, 38039, Kayseri, Turkey
Supporting Information
| Sr. No. | Table of contents | Page No. |
| --- | --- | --- |
| 1 | MD animation video S1 and S2 of compounds 5c and 5f with CYP51 | 2-3 |

## Slide 2
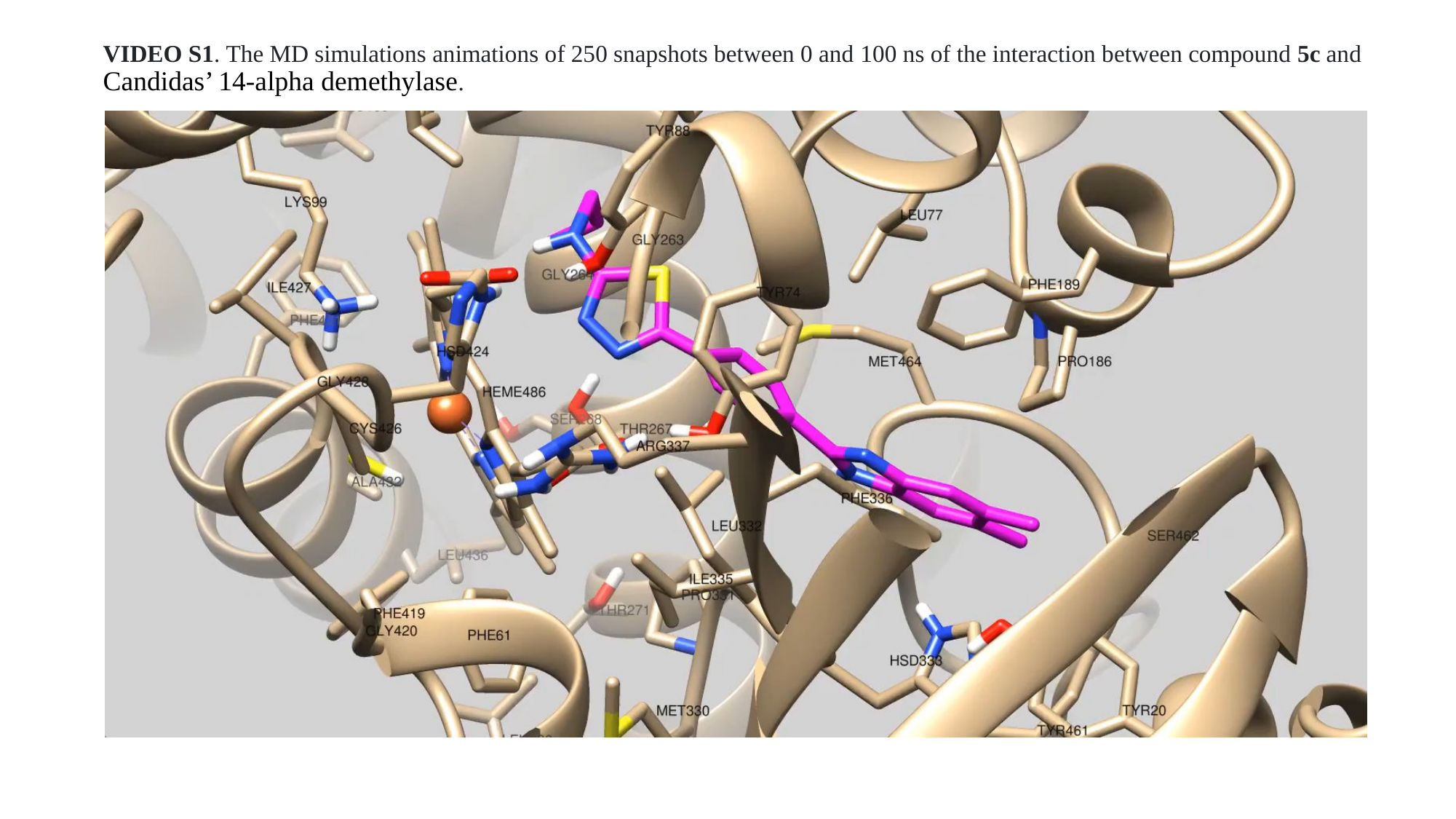

# VIDEO S1. The MD simulations animations of 250 snapshots between 0 and 100 ns of the interaction between compound 5c and Candidas’ 14-alpha demethylase.

## Slide 3
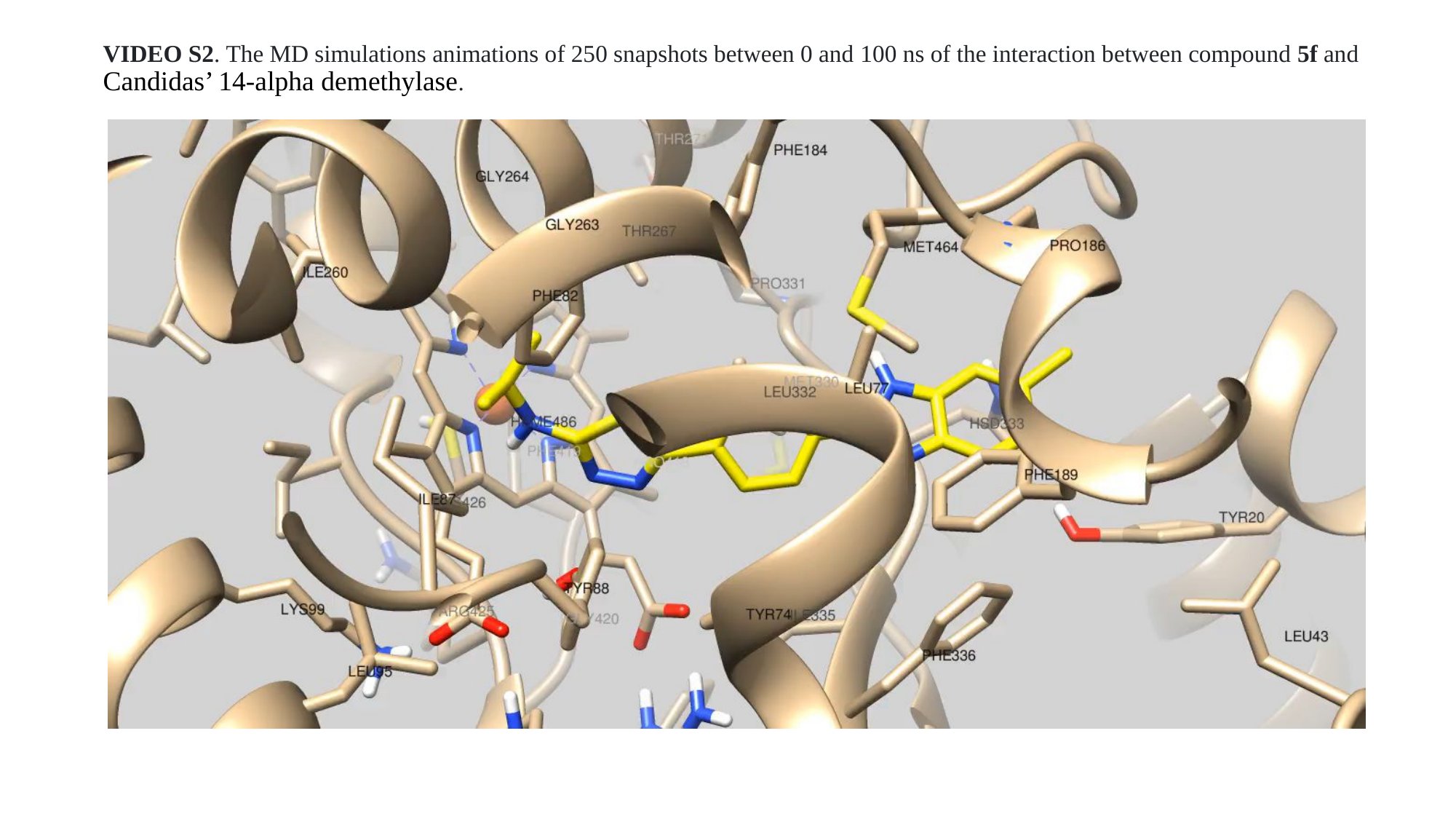

# VIDEO S2. The MD simulations animations of 250 snapshots between 0 and 100 ns of the interaction between compound 5f and Candidas’ 14-alpha demethylase.
